# Supplementary material for: Leishmania major chromosomes are replicated from a single high-efficiency locus supplemented by thousands of lower efficiency initiation events
Source: Cell Rep. 2025 Aug 5;44(8):116094. doi: 10.1016/j.celrep.2025.116094 (PMC12890776; doi:10.1016/j.celrep.2025.116094)
Supplement: Document S1. Figures S1–S5 [file mmc1.pdf]

Cell Reports, Volume *44*

Supplemental information

Leishmania major chromosomes are replicated from a single high-efficiency locus supplemented by thousands of lower efficiency initiation events

Jeziel D. Damasceno, Gabriel L.A. Silva, Catarina A. Marques, Marija Krasilnikova, Craig Lapsley, Dario Beraldi, and Richard McCulloch

Figure S1

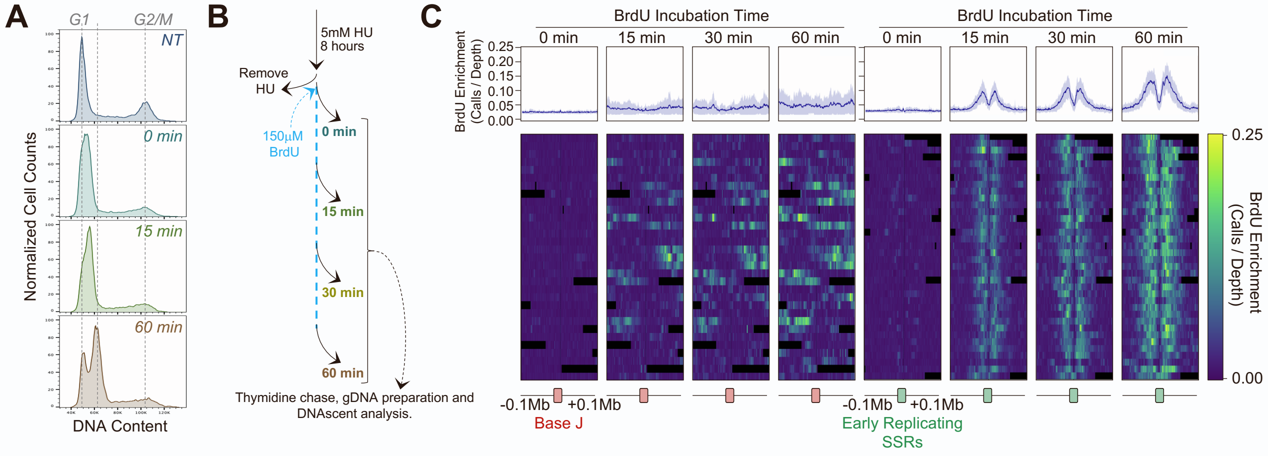

Figure S1. Testing incorporation of BrdU into *Leishmania major* promastigote nuclear DNA, related to Figure 1. A) Exponentially growing *L. major* promastigotes were left untreated (NT) or incubated with HU for 8 hrs to arrest cell cycle, transferred into fresh HU-free medium and collected at the indicated later time points. FACS analysis was used to assess DNA content profile. **B)** Schematic of the experimental approach used for analysis show in C. Exponentially growing *L. major* promastigotes were treated with HU for 8 hrs, then transferred into fresh HU-free medium containing 150 µM BrdU and collected 0, 15, 30 and 60 min later. For each sample, 1 mM thymidine was added for 1 hour, then high molecular weight DNA was extracted and subjected to Oxford Nanopore Technologies sequencing. **C)** Summary plots (top) and colormaps (bottom) comparing BrdU calls density (as normalised by the sequencing depth) between regions of base J accumulation and early replicating SSRs.

Figure S2

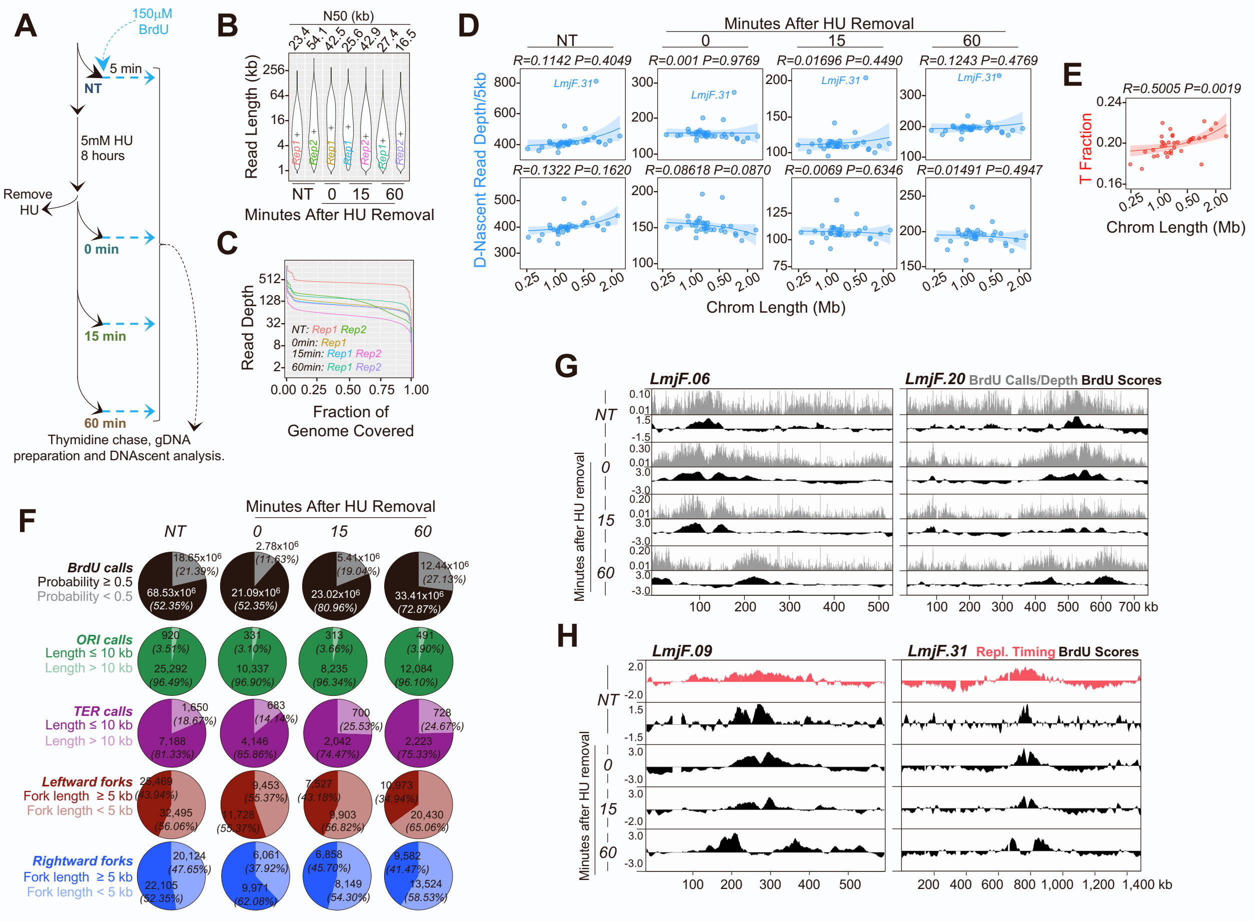

Figure S2. Comparing sequencing yield and BrdU incorporation profiles between NT and HU treated samples, related to Figure 1. A) Schematic of the experimental approach used for all the remaining analysis presented in main and supplementary figures. Exponentially growing *L. major* promastigotes were treated with HU for 8 hrs, then moved into fresh HU-free medium. Cells were collected 0, 15 and 60 min later, then incubated with 150 μM BrdU for 5 minutes followed by 1 mM thymidine chase for 1 hour. High molecular weight DNA was extracted and subjected to Oxford Nanopore Technologies sequencing and analysis with DNAscent. **B)** and **C)** Read length distribution and genome coverage, respectively, from the indicated replicates (Rep) and condition. **D)** and **E)** Simple linear regression analysis comparing average read depth or T content, respectively, from each chromosome with its length. In G, chromosomes 31, which is estimated to be tetraploid, was either included (top) or excluded (bottom) from the analysis. Shaded areas represent 95% confidence intervals. *R* and *P* values are indicated at the top of each panel. **F)** Proportion of retained (darker coloured) and discarded (light coloured) of the indicated features after filtering based on read length (>10kb) and mapping quality (>20). **G)** Snapshots showing distribution BrdU along the entire indicated chromosomes in NT cells and after HU synchronisation. In grey, BrdU signal expressed as the number of calls normalised by the sequencing depth. In black, BrdU score which is the z-scores of calls normalised by the read depth. **H)** Snapshots showing BrdU scores (black) in the entire indicated chromosomes in NT cells and after HU synchronisation. Top track (salmon), DNA replication timing profile as determined by MFA-seq in NT cells.

Figure S3

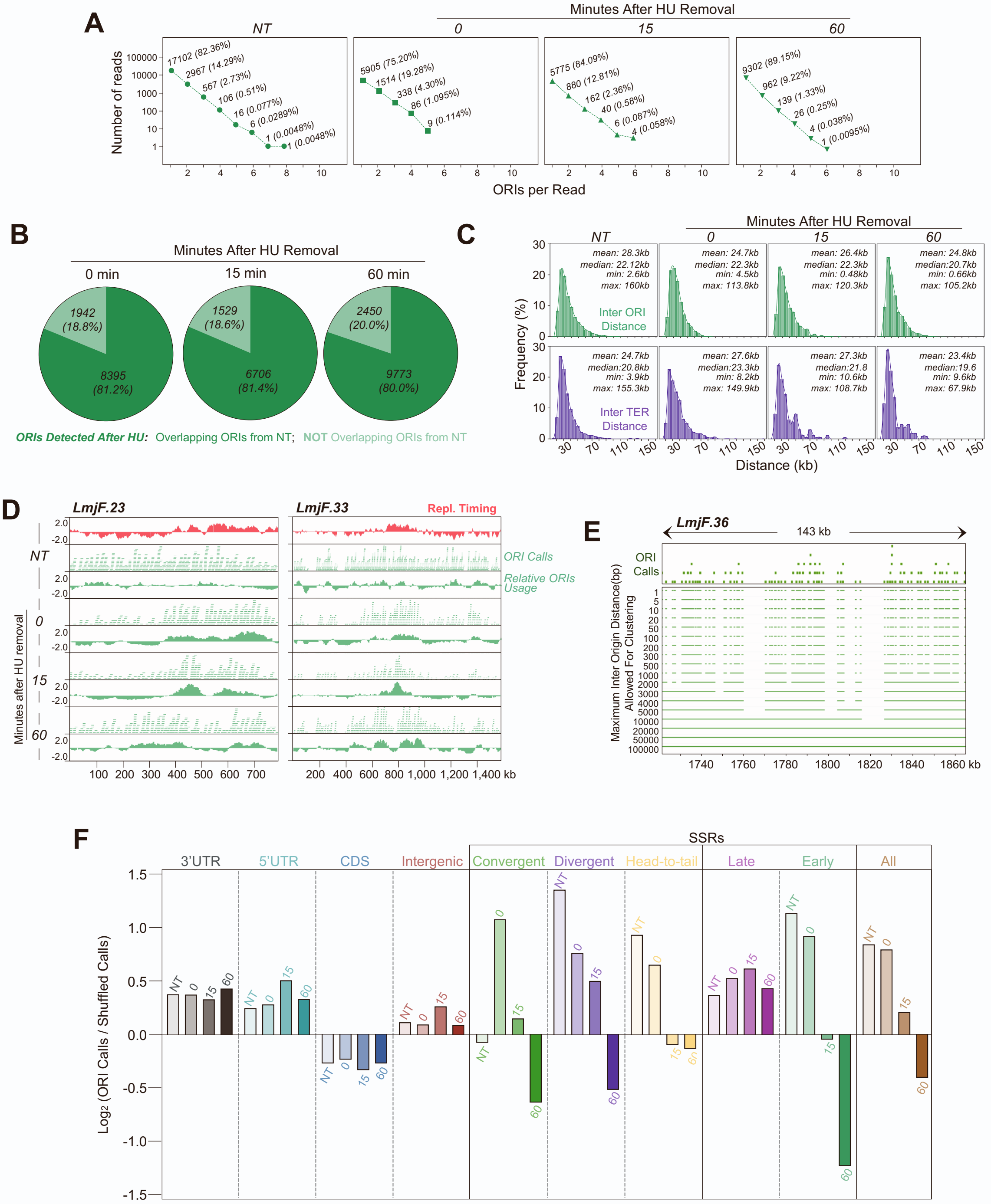

Figure S3. Genome-wide distribution of ORIs detected by DNAscent, related to Figure 2. A) Number and proportion of reads with the indicated number of ORIs from NT and HU synchronised cells. **B)** Genome-wide quantification of the overlap between ORIs from HU treated cells and NT cells. **C)** Frequency distribution of distances between pairs of ORIs or TERs detected in the same single DNA molecule from NT and HU synchronised cells. **D)** Snapshots showing ORIs distribution (green) in the entire indicated chromosomes in NT and HU synchronised cells. ORI calls as detected at the single molecule level is shown as the first green track of each condition. Relative ORIs usage expressed as the z-scores of the number of calls in 5 kb windows normalised by the sequencing depth is shown as the second green track of each condition. Top track (salmon), DNA replication timing profile as determined by MFA-seq in NT cells. **E)** Clustering of DNAscent ORI calls. A window of 143 kb from chromosome 36 showing clusters generated when allowing the indicated maximum distance between ORI calls. Each horizontal green bar indicate an individual cluster. The extension of each green bar is referred to as cluster width. **F)** Distribution of DNAscent ORI calls in the indicated annotated genomic features from *L. major* in NT cells and at the indicated time points after HU removal.

Figure S4

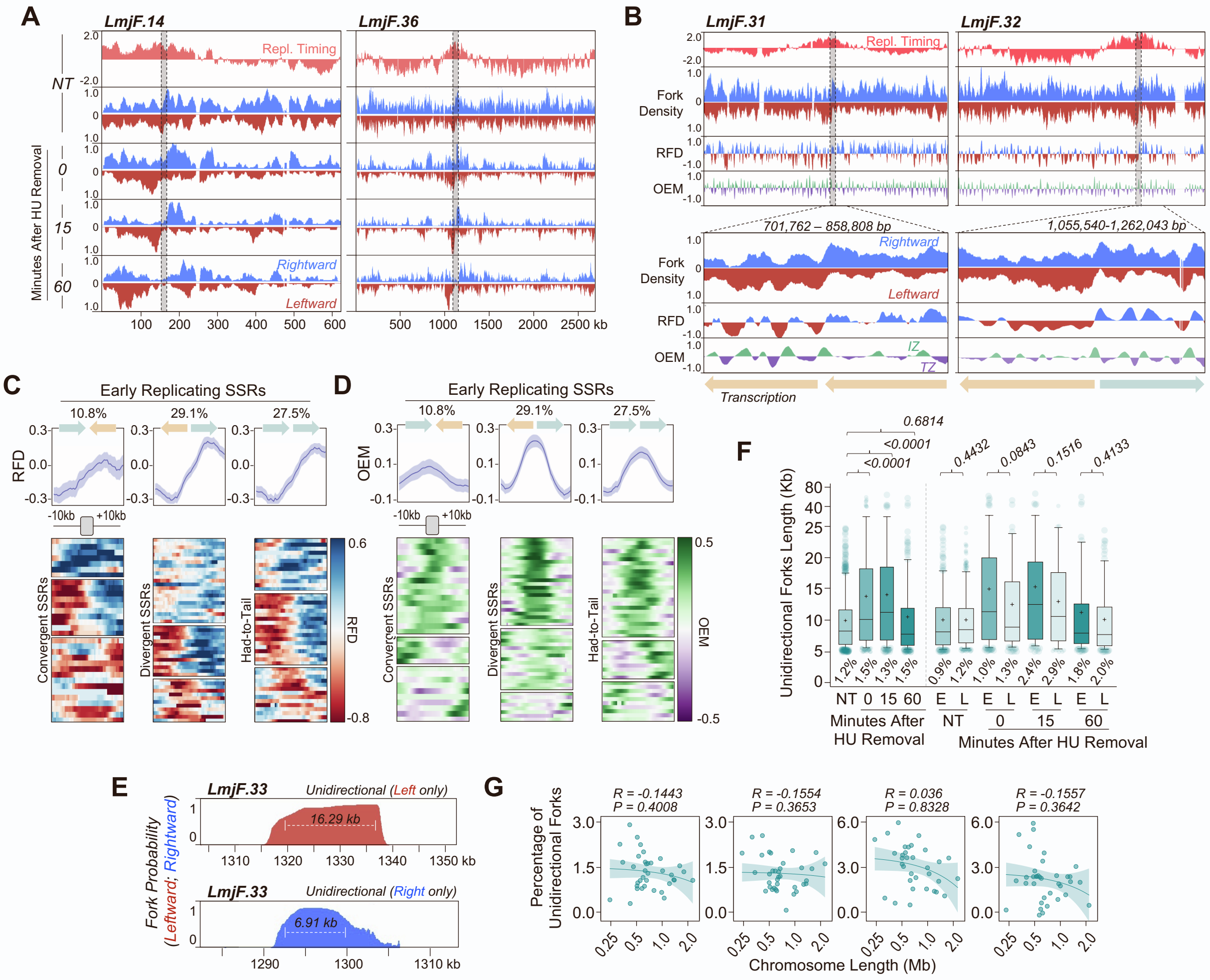

Figure S4. Analysis of DNA replication forks detected by DNAscent, related to Figure 4. A) Snapshots showing density of normalised leftwards (red) and rightwards (blue) moving forks in the entire indicated chromosomes in NT and HU treated cells. Top track (salmon), DNA replication timing profile as determined by MFA-seq in NT cells. Grey vertical bar, position of the single early replicating SSR. **B)** Snapshots showing normalised density of leftwards (red) and rightwards (blue) moving forks and the corresponding RFD and OEM profiles for the entire indicated chromosomes in NT cells. Magnification around the single early replicating SSR is displayed. Arrows at the bottom indicate transcription direction in the PTUs flanking the early replicating SSRs. **C)** and **D)** Summary plots (top) and colourmaps (bottom) comparing RFD and OEM profiles, respectively, around the indicated group of SSRs in NT cells. SSRs were grouped according to the transcription direction from their flanking PTUs: convergent SSRs, where transcription from upstream and downstream PTUs terminates; divergent SSRs, where transcription of upstream and downstream PTUs initiates; head-to-tail SSRs, where transcription from the upstream and downstream PTUs terminates and initiates, respectively. The fraction of SSRs that are predicted by MFA-seq to be early replicating in each group is shown at the top. **E)** Representative Nanopore individual reads showing reads in which only leftwards and rightwards moving forks were detected. Leftwards and rightwards moving replication forks probabilities are indicated as red and blue, respectively. White dotted horizontal lines indicate the position of replication forks calls. **F)** Comparing the length of unidirectional forks between NT and HU treated cells. The percentages of unidirectional forks relative to the total number of left and right moving forks is indicated bellow each box plot. E and L, early and late replicating compartments. *P* values are indicated at the top. Statistical test, Kruskal-Wallis. **G)** Simple linear regression between chromosome length and the average percentage of unidirectional forks in each chromosome. Shaded areas represent 95% confidence intervals. *R* and *P* values are indicated at the top of each panel.

Figure S5

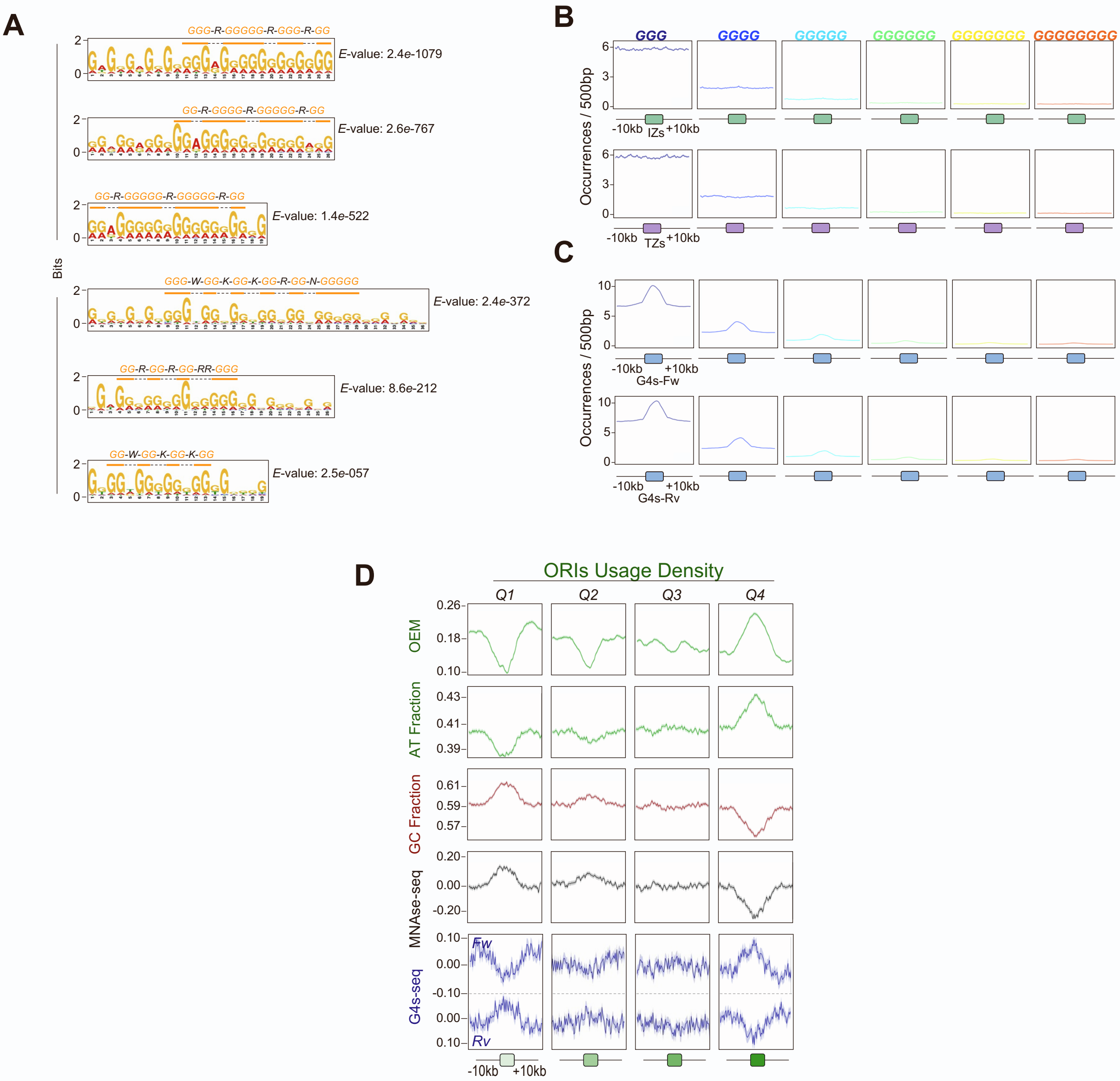

Figure S5. Sequence and chromatin content analysis in replication initiation zines, related to Figure 5. A) MEME sequence motif analysis of G4s overlapping IZs. G4s sequences from each motif is shown at the top with G tetrads in yellow and loops in black. All identified motifs fall into the $G_{2+}L_{1-12}$ G4s category, detectable mainly upon stabilisation (*Marsico et al, 2019*). **B)** Metaplots showing global profiles of poly(dG:dC) of the indicated lengths around all IZs and TZs from NT cells. **C)** Same as in B, but around all G4s found within IZs. **D)** Metaplots showing global OEM, AT and GC content, MNase-seq and G4-seq profiles around genome bins (5kb) grouped according to their ORI usage density from NT cells (Q1, lower usage; Q4, higher usage).
