# Supplementary material for: Leishmania major chromosomes are replicated from a single high-efficiency locus supplemented by thousands of lower efficiency initiation events
Source: Cell Rep. 2025 Aug 5;44(8):116094. doi: 10.1016/j.celrep.2025.116094 (PMC12890776; doi:10.1016/j.celrep.2025.116094)
Supplement: Document S2. Article plus supplemental information [file mmc2.pdf]

Cell Reports

Article

Leishmania major chromosomes are replicated from a single high-efficiency locus supplemented by thousands of lower efficiency initiation events

Graphical abstract

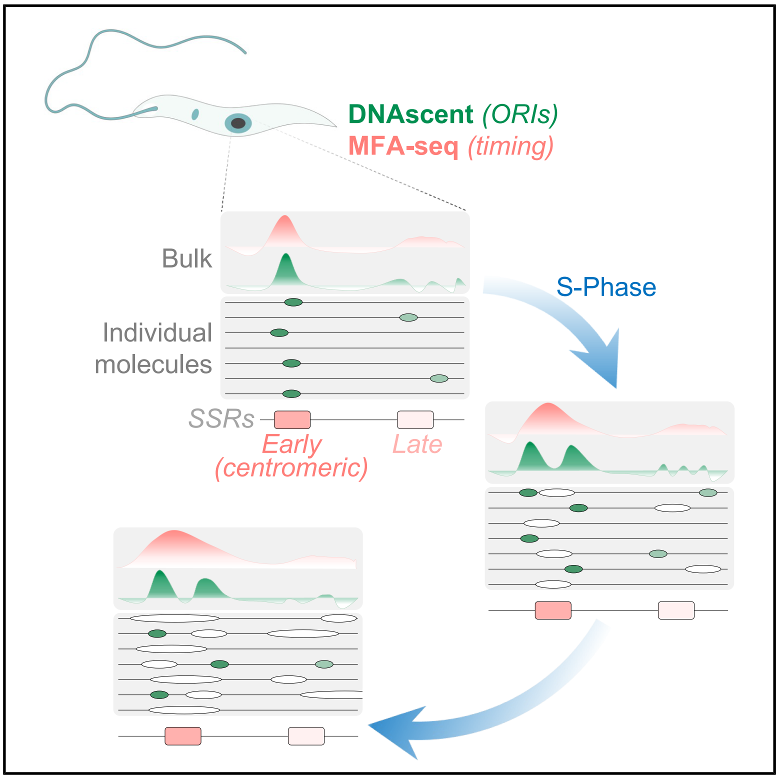

Highlights

- Single-molecule mapping of DNA replication in the protozoan parasite *Leishmania major*
- DNAscent confirms predictions of a single, pre-eminent initiation locus in each chromosome
- DNAscent reveals highly abundant, stochastic initiation events across chromosomes
- Bimodal DNA replication initiation explains *L. major* genome replication timing and variation

Authors

Jeziel D. Damasceno, Gabriel L.A. Silva, Catarina A. Marques, Marija Krasilnikova, Craig Lapsley, Dario Beraldi, Richard McCulloch

Correspondence

jeziel.damasceno@glasgow.ac.uk (J.D.D.), richard.mcculloch@glasgow.ac.uk (R.M.)

In brief

Mapping DNA replication in cell populations can miss low-efficiency events and cell-to-cell heterogeneity. Using single-molecule mapping, Damasceno et al. reveal a program of DNA replication in *Leishmania major* comprising high-efficiency DNA initiation at a single locus in each chromosome supplemented by widespread and stochastic low-efficiency initiation.

Damasceno et al., 2025, Cell Reports *44*, 116094
August 26, 2025 © 2025 The Author(s). Published by Elsevier Inc.
https://doi.org/10.1016/j.celrep.2025.116094

CellPress

Cell Reports

CellPress
OPEN ACCESS

Article

Leishmania major chromosomes are replicated from a single high-efficiency locus supplemented by thousands of lower efficiency initiation events

Jeziel D. Damasceno,[1,*] Gabriel L.A. Silva,[1] Catarina A. Marques,[1] Marija Krasilnikova,[1] Craig Lapsley,[1] Dario Beraldi,[1] and Richard McCulloch[1,2,*]

[1]The University of Glasgow Centre for Parasitology, The Wellcome Centre for Integrative Parasitology, University of Glasgow, School of Infection and Immunity, Sir Graeme Davies Building, 120 University Place, G12 8TA Glasgow, UK

[2]Lead contact

*Correspondence: jeziel.damasceno@glasgow.ac.uk (J.D.D.), richard.mcculloch@glasgow.ac.uk (R.M.)

https://doi.org/10.1016/j.celrep.2025.116094

SUMMARY

DNA replication initiates at genome sites termed origins. Previous origin mapping approaches in the populations of the protozoan parasite *Leishmania major* were discordant, suggesting either a single origin per chromosome or 200-fold more origins. To reconcile these data and fully understand DNA replication dynamics, we have applied DNAscent, an assay that detects patterns of 5-bromodeoxyuridine (BrdU) incorporation in individual long-read DNA molecules. We confirm the pre-eminence of a single locus of DNA replication initiation in each chromosome and reveal a much larger number of lower-efficiency DNA replication initiation events whose abundance is greater as chromosome size increases. Each initiation site is a region of high AT content, increased G-quadruplex levels, lowered chromatin occupancy, and reduced levels of nascent RNA. Finally, we show that all DNA replication initiation results in mutagenesis. This work reveals a bimodal strategy for DNA replication programming in *Leishmania* that drives replication timing and sequence variation.

INTRODUCTION

Propagation of life depends upon the successful duplication of an organism's genome. In cellular organisms, DNA replication initiates at defined sites in the genome termed origins,[1] which in eukaryotes are designated by the binding of the origin recognition complex (ORC).[2] Unlike in bacteria and some archaea, where the whole genome is replicated from a single origin, each eukaryotic chromosome is replicated from multiple origins. This increase in origin number is associated with increased complexity in DNA replication organization. With the exception of *Saccharomyces cerevisiae* and related yeasts,[3] origins in eukaryotes differ from those in prokaryotes in that they are not conserved DNA sequences but are defined by sequence-independent genomic features. In addition, though origins are designated by ORC binding in the G1 phase of the cell cycle and then activated in S phase, not all designated origins are activated. Moreover, there is a temporal order to the time in S phase when origins are activated,[4,5] and, in the case of multicellular organisms, both the number and location of origins can vary during development and differentiation.[1] Due to such complexity, mapping DNA replication origins in eukaryotic cells is technically challenging, and results from different techniques are often discordant.[6]

To detect the origins, most approaches rely on mapping the DNA replication machinery or capturing replicative DNA synthesis in populations of cells, with the potential that only frequently used origins are detected and cell-to-cell heterogeneity is overlooked.[1,6,7] These limitations have been addressed by the recent development of genome-wide single-molecule DNA replication mapping[8–11] and single-cell DNA replication sequencing[12–14] approaches, which have revealed previously undetected origins and informed on DNA replication timing. In *S. cerevisiae*, two related approaches (DNAscent and Fork-seq/NanoForkSpeed)[8,9,15] used Oxford Nanopore Technologies (ONT) sequencing to detect the incorporation of the thymidine analog 5-bromodeoxyuridine (BrdU) into newly replicated DNA, revealing that around 10%–20% of DNA initiation events do not localize to previously reported origins.[8,9] Whether these newly predicted initiation sites correspond to a predicted 15% of minichromosome maintenance helicase (MCM)-binding sites that do not localize with sequence-conserved origins[16] awaits testing. In the larger genome of humans, ~80% of the origins detected by DNAscent do not overlap with origins previously detected by population-level analyses,[17] perhaps consistent with a model of stochastic DNA replication initiation derived from optical replication mapping.[10] Single-cell DNA replication sequencing methods, such as scRepli-seq, measure copy-number variation (CNV) between replicating and non-replicating DNA and, instead of mapping origin location, describe replication timing domains across S phase.[13,14] These approaches have been used in human and mouse cells and, overall, reveal conservation of DNA replication timing organization in individual cells and equivalent populations,[13,14] although timing domains differ between cell types.[12,14]

© 2025 The Author(s). Published by Elsevier Inc.
This is an open access article under the CC BY license (http://creativecommons.org/licenses/by/4.0/).

Thus, despite origin activation being stochastic, the mammalian DNA replication timing program is spatially well defined, perhaps to ensure maximal efficiency of genome duplication and coordination with other processes, such as transcription.

Protozoans provide much of the diversity in the eukaryotic domain,[18,19] and only relatively recently has DNA replication been examined in a few select organisms in this grouping, with new insights emerging in origin usage and DNA replication programming. For instance, two nuclei are found in the single cells of *Giardia lamblia*[20] and *Tetrahymena thermophila*,[21] with highly distinct genome organization and function found in the distinct nuclei of the latter. In both cases, DNA replication dynamics appear to differ between the nuclei, but no work has mapped origins genome-wide, and so how potentially distinct genome duplication activities are organized within a single cell remains unclear. The life cycle of the malaria parasite *Plasmodium* is complex, containing four stages with distinct replicative strategies: hepatic and erythrocytic schizogony, gametogenesis, and sporogony.[22] Recently, two studies have combined chromatin immunoprecipitation sequencing of ORC subunits and NanoForkSpeed or DNAscent to detect the origins in *Plasmodium falciparum* undergoing erythrocytic schizogony,[23,24] a process in which the parasite undergoes multiple rounds of asynchronous DNA replication and nuclear division without cytokinesis, resulting in a multinucleated schizont. Surprisingly, no clear consensus emerged from the two studies, perhaps suggesting that DNA replication during schizogony has unanticipated complexities. Arguably, the most advanced understanding of protozoan DNA replication has emerged within the kinetoplastids,[25,26] a ubiquitous grouping of flagellated organisms that includes both human and animal parasites of clinical and economic importance. DNA replication dynamics has been described and compared in three kinetoplastid parasites (see Devlin et al., Marques and McCulloch, da Silva et al., Tiengwe et al., Rocha-Granados and Klingbeil, and Damasceno et al.[27–32] for reviews): *Trypanosoma brucei*,[33–35] *Trypanosoma cruzi*,[36,37] and *Leishmania* sp.[38–40] Despite each of these parasites using a common, highly unusual form of gene expression where nearly every gene is expressed from a polycistronic transcription unit (PTU),[41] and each genome sharing considerable synteny,[42,43] population-level DNA replication analyses suggest pronounced differences, in particular, between *T. brucei* and *Leishmania*.[32]

The genome of *T. brucei* is primarily housed in 11 diploid "megabase" chromosomes, each of which contains a highly transcribed core and two largely transcriptionally silent subtelomeres, which are variable in content between strains[44] and mainly harbor thousands of genes encoding variant surface glycoproteins.[45–47] To date, *T. brucei* DNA replication has only been mapped genome-wide using population-level marker frequency analysis sequencing (MFA-seq; equivalent to sort-seq[48] in yeast).[33–35,49,50] This approach predicts origins within the megabase chromosome cores at the boundaries ("strand switch regions," SSRs) of ~25% of PTUs. Consistent with these loci being origins, one subunit of *T. brucei* ORC[51–53] has been shown to bind to all SSRs, suggesting that only a subset of ORC-binding sites is activated to initiate DNA replication in early S phase,[33] with such origin selection being invariant between distinct life cycle stages and parasite strains.[34] Among these MFA-seq-predicted origins, those co-localizing with centromeres are the earliest replicating.[33,34] More recent MFA-seq mapping suggests that most of the subtelomeric compartment of the *T. brucei* genome is late replicating and more unstable than the core.[35] Altogether, these data reveal incompletely explored links between DNA replication initiation, transcription, chromosome segregation, and genome stability in this organism.[33–35,49,50]

Equivalent MFA-seq analysis in two *Leishmania* species, *L. major* (36 chromosomes) and *L. mexicana* (34 chromosomes), revealed a striking difference to *T. brucei*: only a single MFA-seq peak indicative of S phase DNA replication initiation could be detected in each chromosome.[38] As no study has mapped ORC in either *Leishmania* genome, it is premature to say that the MFA-seq signal in each chromosome represents an origin, but a range of observations is consistent with such a suggestion: first, as in *T. brucei*, each MFA-seq signal centers on an SSR; second, mapping the binding of the kinetochore subunit KKT1 is consistent with the single MFA-seq SSR in each chromosome being a centromere,[54] which is coincident with the earliest acting origins in *T. brucei*[33]; third, ~40% of the *Leishmania* MFA-seq SSRs are syntenic with origin-active SSRs in *T. brucei*[38]; and finally, the single MFA-seq peak in each chromosome overlaps with highly localized changes in base composition skews[39] as well as with increased mutagenesis,[40] each feature being consistent with the sites of frequent DNA replication initiation. Beyond these data on the putative *Leishmania* origin-active SSRs, average chromosome replication timing in *L. major* is unusual in that it correlates with chromosome length, with larger chromosomes being replicated later than smaller ones.[39] Such timing may be consistent with limiting DNA replication initiation to just a single locus in each chromosome, but such programming appears insufficient to allow duplication of the largest ~40% of the chromosomes during S phase.[38] At least in part, this limitation may be overcome by DNA replication activity proximal to the telomeres of each *L. major* chromosome that is detectable outside S phase.[39] Nonetheless, two different approaches have suggested that MFA-seq may not detect all DNA replication activity in *Leishmania*. DNA combing has detected >1 site of DNA replication initiation in single *Leishmania* DNA molecules,[55,56] and short nascent strand sequencing (SNS-seq) has mapped >5,000 putative origins throughout the *L. major* genome, with only limited overlap with MFA-seq mapping.[57] These discrepancies may arise from technical limitations in each of these methodologies. MFA-seq analyzes CNV in replicating versus non-replicating cells, and therefore mainly determines the timing of DNA replication, with limited spatial resolution to pinpoint origins. DNA combing, as reported to date in *Leishmania*, is low-throughput and does not localize where in a chromosome, or even in what genome, DNA replication events are detected; indeed, it cannot exclude the possibility that the multi-origin molecules described are derived from abundant extrachromosomal elements.[58] Finally, SNS-seq mapping is known to be susceptible to G-quadruplex (G4) impediments,[59] which may be a particular concern given the high prevalence of G4s in the *L. major* genome.[60]

Irrespective of technical considerations, the huge discrepancies between these studies in the estimates of origin number and location raise questions about how *Leishmania* DNA

CellPress
OPEN ACCESS

replication is programmed, including why MFA-seq portrays such a stark difference relative to *T. brucei*. Here, we address these questions using DNAscent, a deep learning-based approach developed by Muller et al.[8,61] that, by detecting BrdU in newly replicated DNA on long, single-molecule Nanopore sequence reads, provides high-precision mapping of DNA replication fork dynamics, allowing the detection of DNA replication initiation and termination sites. Using this approach, we show that the duplication of the *L. major* genome may be based on two DNA replication initiation processes: the single MFA-seq peak in each chromosome represents a locationally invariant, pre-eminent locus at which DNA replication initiates at high frequency at the onset of S phase, and this activity is supported by much more numerous, stochastic DNA replication initiation events that are distributed across every chromosome. We provide evidence that chromosome length-related DNA replication timing in *L. major* is reflected in differential reliance on stochastic initiation events, which localize to regions with high AT content, increased G-quadruplex levels, and lower chromatin occupancy. Finally, we show that the DNA replication program of *L. major* relates to the patterns of genome variation.

RESULTS

BrdU detection with DNAscent confirms pre-eminent DNA replication initiation at a single locus in each *L. major* chromosome

DNAscent relies on the detection of BrdU in DNA molecules through nucleoside analog signal currents during Nanopore sequencing (Figure 1A). To establish that detection of BrdU incorporated into the nuclear genome of *Leishmania* is possible (for instance, it is not impeded by the signal from the hypermodified thymidine base, β-D-glucopyranosyloxymethyluracil, also called base J),[62] *L. major* promastigote (insect stage) cells were arrested at the G1/S phase transition of the cell cycle by treatment with 5 mM hydroxyurea (HU) for 8 h (Figure S1A). Next, one sample was collected (no BrdU control), while the remainder of the cells were released from the HU block and grown for 15, 30, or 60 min in the presence of 150 μM BrdU (Figure S1B). This treatment was followed by thymidine chase (1 mM thymidine for 60 min), DNA extraction, DNA sequencing on ONT MinION, and BrdU calling with DNAscentv2.[8,61] Any predicted BrdU signal was barely seen in the no BrdU control cells (0.17% of the reads), while substantial BrdU signal accumulated in the BrdU-labeled cells (1.33%–2.24% of the reads). Metaplots did not show any significant BrdU accumulation overlapping base J-enriched sites (Figure S1C) in contrast to time-dependent BrdU accumulation around centromeric SSRs, consistent with the MFA-seq prediction of a single early-replicating locus in each chromosome.[38,39]

To accurately predict replication fork movement, as well as initiation and termination sites of DNA replication, DNAscent relies on the detection of gradients of BrdU incorporation (Figure 1A).[8,61] We therefore modified the aforementioned experiments, again using *L. major* promastigotes, but this time fixing the length of the BrdU pulse to 5 min (Figure S2A). Since the rate of replication fork movement in *L. major* has been calculated as ∼2.5–2.8 kb/min,[55,57] we expected BrdU-labeled tracts in the range of 12.5–15 kb, which is smaller than the N50 range of 16.5–54.1 kb we recovered from Nanopore sequencing (Figures S2B and S2C). For this new experimental setup, we again arrested the cell cycle with HU at the G1/S phase transition, but now collected cells at 0, 15, and 60 min after HU release and then labeled them with 150 μM BrdU. In addition, we labeled cells with 150 μM BrdU for 5 min without any HU treatment, allowing us to compare patterns of BrdU incorporation, and thereby DNA replication dynamics, in an unsynchronized population relative to the early stages of S phase (Figure S1A). In all cases, the BrdU pulse was followed by a 1 mM thymidine chase of 60 min.

Visual inspection revealed BrdU signal across all chromosomes in unsynchronized cells, with the most prominent level of BrdU accumulation in each chromosome localizing around the single early-replicating SSR predicted previously by MFA-seq (Figures 1B, S2G, and S2H). This correspondence resembles the overlap between sort-seq and BrdU enrichment measurements of DNA replication timing reported in yeast.[63] After release from HU arrest, this most prominent BrdU signal was detected as two peaks of increasing separation with time, consistent with the bidirectional progression of DNA replication from the SSR (Figures 1B, S2G, and S2H). To test these observations further, we generated metaplots of BrdU signal around the centromeric SSR in each chromosome (36 "early-replicating SSRs") and around all other SSRs ("late-replicating SSRs"; Figure 1C). The consistent levels of BrdU signal were apparent at the MFA-seq-enriched, early-replicating SSR in each chromosome both before and after HU synchronization: whereas BrdU signal was closely focused around the SSR in unsynchronized cells, two BrdU peaks of very similar amplitude and width were detected flanking the SSRs in the HU-synchronized cells, with the distance between the peaks increasing over time after release from HU arrest (most distal in the 60-min sample). Furthermore, the density of left- and right-moving DNA replication forks exhibited symmetrical accumulation around the early-replicating SSRs in asynchronous cells and progressively separated from each other post HU release, mirroring the BrdU signals (Figures 1D and S4A). In contrast, there was no such BrdU accumulation or replication fork enrichment at the late-replicating SSRs, with or without HU synchronization. Taken together, these data indicate highly coordinated initiation of DNA replication in early S phase concentrated at a single SSR in each chromosome.

Using MFA-seq data, we have previously noted a DNA replication program in *L. major* where average chromosome duplication timing is size dependent (Figure 1E, left panel).[28,38,39,64] To test if DNAscent predicts the same organization of DNA replication, linear regression analysis was conducted on the BrdU density data, which revealed a significant correlation between chromosome size and average BrdU signal, with smaller chromosomes displaying higher BrdU levels than larger chromosomes in non-synchronized cells (Figure 1E, panel NT, colored black). An even stronger correlation was observed in synchronized cells, consistent with a greater extent of duplication of smaller chromosomes in early S phase (Figure 1E, panels 0, 15, and 60, colored black). Importantly, this effect cannot be attributed to systematic biases in read depth (Figure S2D) or base T content (Figure S2E) disproportionately affecting smaller chromosomes.

Taken together, these data show that DNAscent analysis of long-read Nanopore sequencing of BrdU-labeled DNA is not

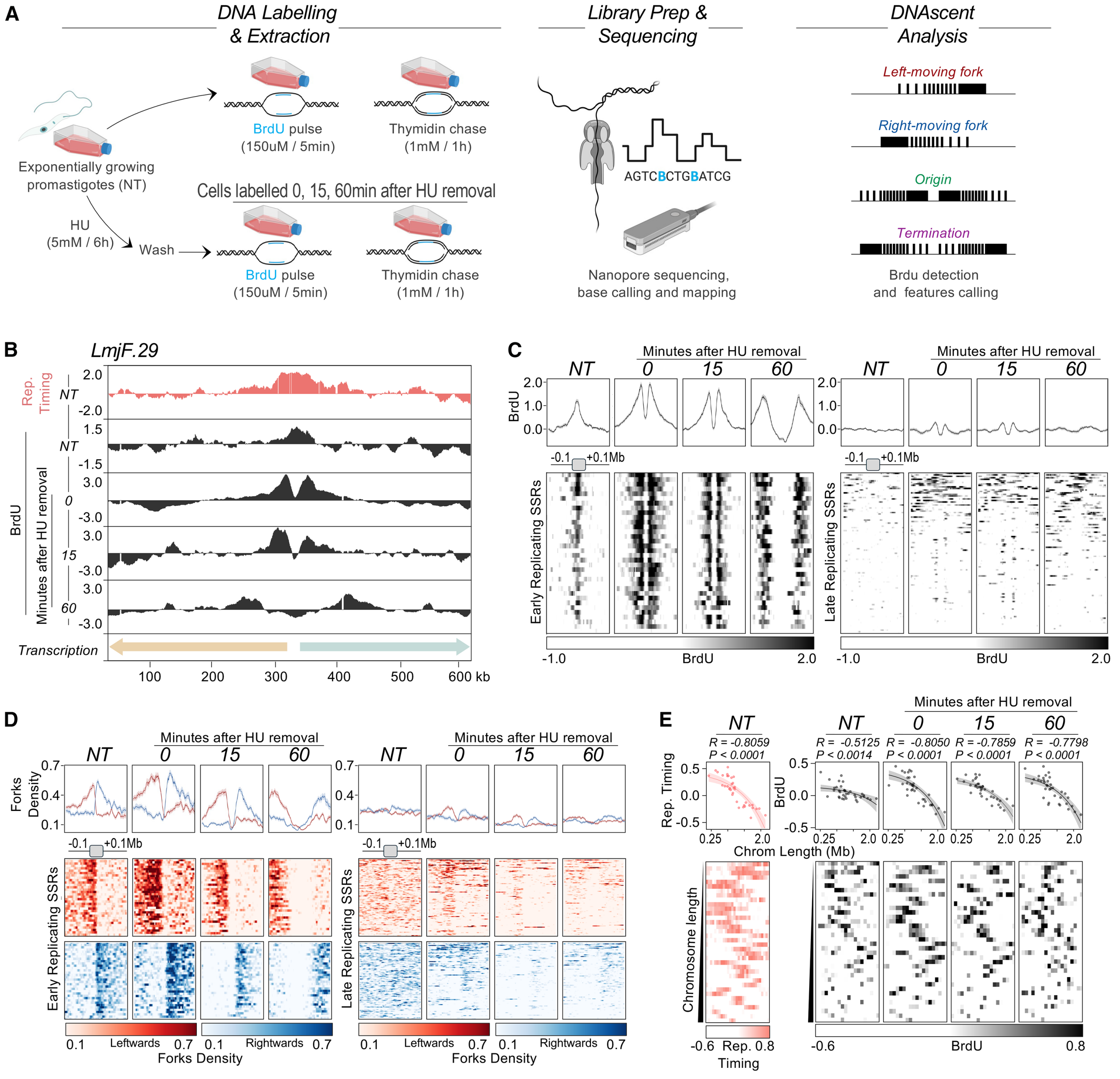

Figure 1. Mapping BrdU incorporation profiles with DNAscent confirms centromeric SSR-driven early DNA replication initiation and chromosome size-associated DNA replication timing

(A) Schematic of the experimental approach (see also Figure S2A). Exponentially growing *L. major* promastigotes (NT) were labeled with BrdU for 5 min (see STAR Methods), followed by a thymidine chase for 1 h. The same labeling conditions were also used 0, 15, and 60 min after parasites were released from cell-cycle arrest at G1/S with HU (Figure S1A). High-molecular-weight DNA was extracted and subjected to ONT sequencing, and data were analyzed with DNAscentv2.

(B) Snapshot showing BrdU scores (black) around a representative early-replicating SSR in *L. major* chromosome 29 in NT cells and 0, 15, and 60 min after HU removal. Top track (salmon), DNA replication timing profile determined by MFA-seq in NT cells.

(C) Summary plots (top) and colormaps (bottom) comparing BrdU score profiles around all early-replicating SSRs (left) and all late-replicating SSRs (right), in NT- and HU-synchronized cells.

(D) Summary plots (top) and colormaps (bottom) comparing density profiles of leftward (red) and rightward (blue) moving DNA replication forks around all early-replicating SSRs (left) and all late-replicating SSRs (right), in NT- and HU-synchronized cells.

(E) Colormaps (lower) showing the replication timing profile of all chromosomes determined by MFA-seq in NT cells (left, salmon) and BrdU score profiles of all chromosomes (right, black) in NT- and HU-synchronized cells. Top, simple linear regression analysis between chromosome length and average MFA-seq signal or average BrdU scores. Shaded areas represent 95% confidence intervals.

R (correlation coeficient) and *p* values are indicated at the top of each panel.

CellPress
OPEN ACCESS

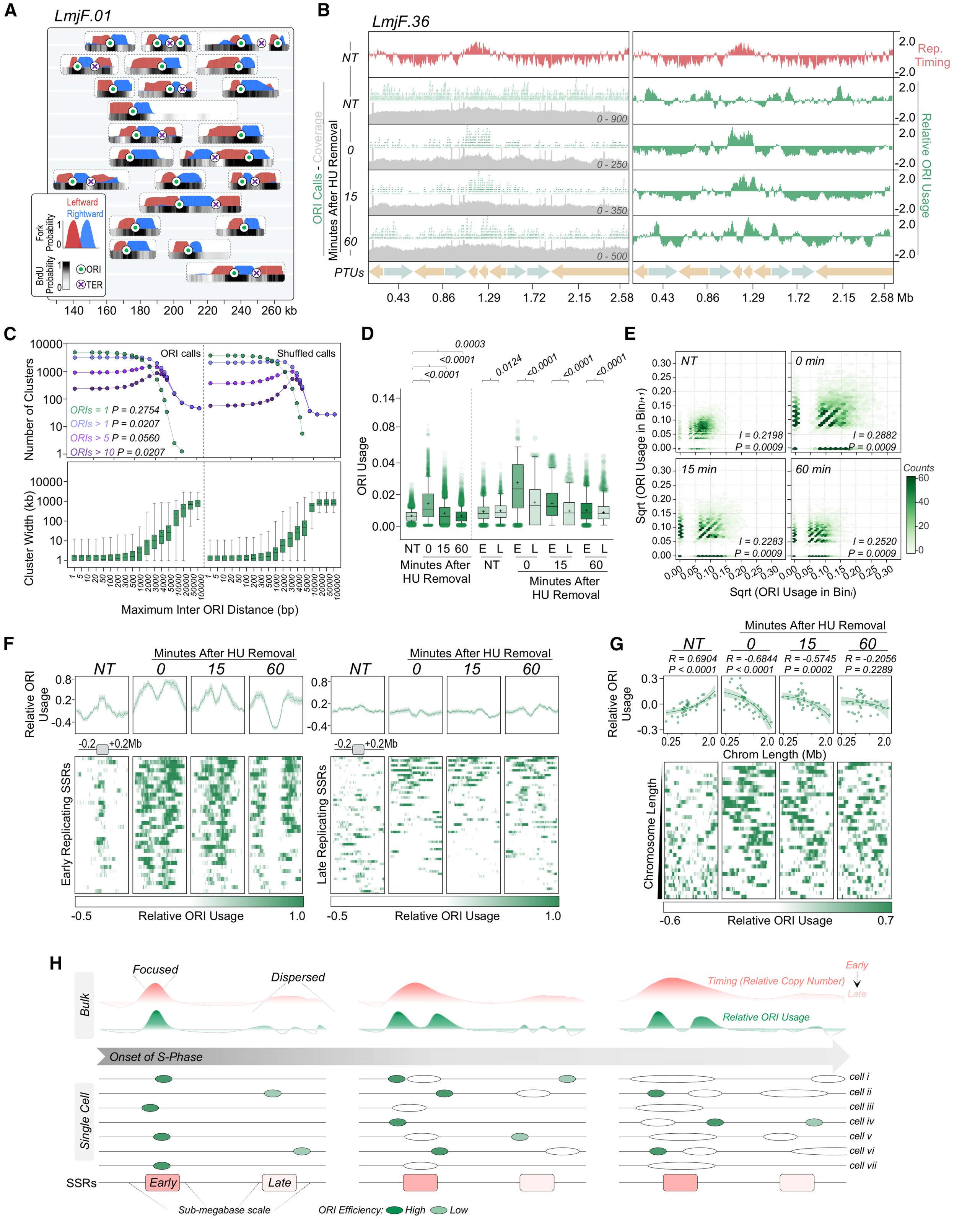

(legend on next page)

only feasible in *L. major* but validates the findings of population-level MFA-seq mapping: in early S phase, there is coordinated initiation of DNA replication from a single locus at each chromosome, which progresses bidirectionally toward chromosome ends and which may dictate chromosome size-dependent replication timing.

DNAscent reveals abundant, previously undetected initiation events in each *L. major* chromosome

An advantage of DNAscent[8,61] or variants[9,11,15,65] over population-level mapping approaches lies in their higher sensitivity, as they can predict DNA replication initiation and termination sites based on fork direction determined from BrdU gradients (Figure 1A) on single DNA molecules that collectively span the genome. Thus, heterogeneous aspects of DNA replication that are lost in population-based methodologies, as signals averaged across many cells and molecules, may be revealed. To explore this, we first visualized individual Nanopore reads, after processing with DNAscent, that mapped to a 130-kb region of chromosome 1 in unsynchronized cells (Figure 2A). Multiple sites of DNA replication initiation and termination were predicted across this small region, with little evidence for common localization. By examining all reads obtained (Figure S3A), DNAscent predicted ∼25,000 initiation sites in non-synchronized cells and ∼8,200–12,100 initiation sites in HU-synchronized cells, a difference likely explained by each HU sample capturing cells in a limited window of S phase (Figure S2F). Nonetheless, by allowing a maximum distance of 1 kb between calls midpoints, ∼80% of initiation sites predicted in synchronized cells were seen in the unsynchronized cells, indicating that DNA replication initiation perturbation due to HU-associated stress is minimal (Figure S3B). Although these predictions will overestimate the total number of DNA replication initiation sites, DNAscent suggests a density of DNA replication initiation events substantially greater than inferences made from DNA combing[55] (∼168 per haploid genome) and from SNS-seq mapping,[56] which predicts ∼5,100. Such a conclusion is consistent with analysis of the distance between DNAscent-predicted initiation sites on individual reads: a median of 20.7–22.3 kb (Figure S3C; range 0.48–160 kb) is at least 3- to 4-fold closer than the ∼72–193 kb inter-origin distance reported by DNA combing.[55,57]

ORIs predicted by DNAscent are widely distributed across the *L. major* genome

The aforementioned data predict the existence of numerous, hitherto undetected DNA replication initiation sites across the *Leishmania* genome. As we do not know if any or all DNAscent-predicted initiation sites correspond to ORC-defined origins, we will refer to them as ORIs. To compare ORI distribution with MFA-seq in unsynchronized and HU-synchronized cells, we determined ORI usage by aggregating all calls from each single molecule and normalizing to sequencing depth (Figures 2B and S3D). In contrast to the clear correspondence between BrdU and fork density with the single MFA-seq peak in each chromosome (Figures 1B–1D), visualization revealed ORIs to be distributed across all chromosomes in unsynchronized cells and not limited to early-replicating SSRs (Figures 2B and S3D). To assess whether ORI distribution was random, we performed a clustering analysis of ORIs across a range of maximum allowed distances between events (Figure S3E) and compared the number and width of clusters to those expected from a random distribution (Figure 2C). Although the observed and shuffled profiles differed statistically, they were visually indistinguishable, suggesting a lack of order to ORI distribution in unsynchronized cells. Consistent with this, only minor deviations between observed and randomized ORI distributions were seen at specific genomic features in unsynchronized cells, though SSRs and gene untranslated regions (UTRs) showed slight ORI enrichment and coding sequences (CDSs) exhibited mild depletion (Figure S3F). In addition, we observed a low correlation

Figure 2. DNAscent shows that origin activation in early S phase is focused on the single centromeric SSR in each chromosome and reveals widespread initiation events whose distribution is chromosome size-dependent

(A) Randomly selected Nanopore individual reads overlapping the indicated region in chromosome 1 are shown: at the bottom of each read, gray to black colormap indicates BrdU probabilities; leftward and rightward moving replication fork probabilities, ranging from 0 to 1 on the *y* axis, are shown in red and blue, respectively; ORIs (between diverging replication forks) and termination sites (TERs, between converging replication forks) are indicated by green circles and purple crosses, respectively.

(B) Snapshots showing ORI distribution across chromosome 36 in NT cells and 0, 15, and 60 min after HU removal. Left, ORI calls, as detected at the single molecule level in green, and in gray, the coverage range is shown for each track. Right, relative ORI usage expressed as the number of calls in 50 kb rolling windows normalized by the sequencing depth, followed by *Z* score transformation. Top track (salmon), DNA replication timing profile as determined by MFA-seq in NT cells. Polycistronic transcription units (PTUs) are shown at the bottom.

(C) Clustering analysis of aggregated ORI calls from NT cells (Figure S3E). The distribution of the number of clusters containing the indicated number of ORI calls (top) and cluster width (bottom) is plotted against the maximum allowed inter-ORI distance for clustering. Shuffled calls, ORI calls after being randomly distributed. *p* values are indicated. Statistical test, Kolmogorov-Smirnov.

(D) Comparing the ORI usage between NT- and HU-treated cells. E and L, early- and late-replicating compartments, respectively. Horizontal line and cross, median and mean, respectively. *p* values are indicated at the top. Statistical test, Kruskal-Wallis.

(E) Hexagonal bins density plot comparing ORI usage between a given bin (Bin_i) and its neighbor (Bin_{i+1}). *I* and *P*, spatial autocorrelation index and *p* value, respectively, as determined by Moran's I autocorrelation test.

(F) Summary plots (top) and colourmaps (bottom) comparing relative ORI usage profile around all early-replicating SSRs (left) and all late-replicating SSRs (right), in NT- and HU-synchronized cells.

(G) Colormaps (lower), relative ORIs usage profile of all chromosomes in NT- and HU-synchronized cells. Top, simple linear regression analysis between chromosome length and average ORI usage density. Shaded areas represent 95% confidence intervals. *R* and *p* values are indicated at the top of each panel.

(H) Schematic illustration of the DNA replication initiation program in *Leishmania*. Top, average replication timing profile (salmon) and ORI usage (green) at the population level. Lower, each horizontal line represents an individual cell within the population. Dark green circles, origins of higher efficiency, which are activated more frequently during each cell cycle. Light green circles, ORIs of lower efficiency, activated less frequently per cell cycle. White circles, bidirectional replication forks. At the onset of S phase, high-efficiency origins are activated at or around the centromeric SSR in each chromosome; in contrast, lower-efficiency ORIs are dispersed across the genome, with increased cell-to-cell variability.

CellPress
OPEN ACCESS

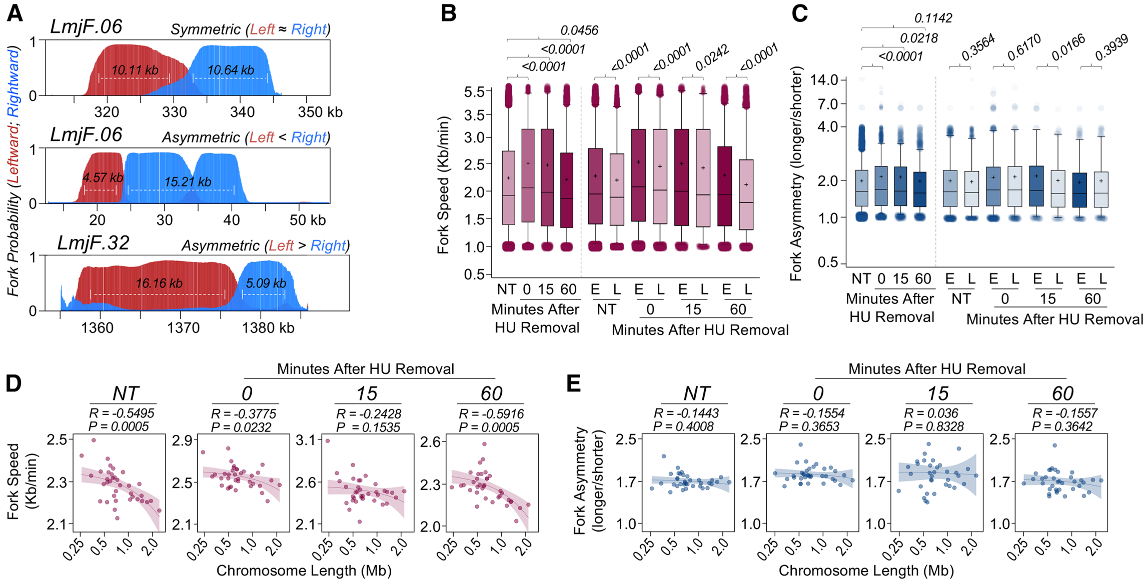

Figure 3. DNAscent reveals variation in fork speed that correlates with DNA replication timing and chromosome length

(A) Representative Nanopore reads showing symmetric and asymmetric replication forks. Leftward and rightward moving replication forks' probabilities are indicated as red and blue, respectively. White horizontal lines indicate the position of replication fork calls. Fork speed was calculated by dividing the length (in kb) of the replication fork calls by the duration of the BrdU pulse (5 min). Fork asymmetry was expressed as the ratio between the longer and the shorter fork calls emerging from the same origins: values close to 1, fork symmetry; values > 1, fork asymmetry.

(B and C) Comparing fork speed (B) and asymmetry (C), respectively, between NT cells and at the indicated time points after HU removal. E and L, early- and late-replicating compartments. Horizontal line and cross, median and mean, respectively. *p* values are indicated. Statistical test, Kruskal-Wallis.

(D and E) Simple linear regression between chromosome length and average fork speed (D) or asymmetry (E), respectively, in each chromosome. Shaded areas represent 95% confidence intervals. *R* and *p* values are indicated.

in ORI usage between adjacent genomic bins in unsynchronized cells (Figure 2E). Altogether, these observations indicate that ORIs represent stochastic sites of DNA replication initiation, without evidence for clustering or pronounced localization across the *L. major* genome.

In contrast to unsynchronized cells, visual inspection suggested that ORI density was substantially increased and localized around the early-replicating SSRs upon cell-cycle arrest, and spread outward 15 and 60 min after release from HU-synchronization (Figures 2B and S3D). Accordingly, ORI usage significantly increased in synchronized cells globally, and most notably in early-replicating genome compartments (Figure 2D). Furthermore, metaplots confirmed a pronounced increase in ORI usage around the 36 early-replicating SSRs in HU-synchronized cells relative to unsynchronized cells, with areas of high ORI density detected at increasing distance upstream and downstream of the SSRs 15 and 60 min after HU release (Figure 2F, see also Figure S3D), an effect comparable to BrdU and fork density mapping (Figures 1C and 1D). Such localized ORI enrichment was not seen at late-replicating SSRs, with or without HU treatment, meaning transcription initiation or termination are not direct determinants of ORI usage. Despite this locally increased ORI usage, the extent of correlation between ORI usage from adjacent genomic bins remained globally unaltered upon HU synchronization (Figure 2E), suggesting ORIs arise rapidly in S phase.

To ask if ORI usage reflects chromosome size-associated replication timing, we plotted average ORI usage across chromosomes (Figure 2G). In unsynchronized cells, a significantly greater ORI density was seen in larger, later replicating chromosomes (Figure 2G). In contrast, immediately after HU release, when most cells are in early S phase (Figure S1A), ORI density was markedly greater on the smaller chromosomes, with such enrichment progressively diminishing in the 15- and 60-min samples (Figure 2G).

Taken together, these data reveal several features of DNA replication programming in *L. major*. First, at the onset of S phase, initiation is predominantly spatially confined to centromeric SSRs, consistent with the single MFA-seq peak in each chromosome (Figure 2H). Second, complete genome duplication is aided by abundant stochastic initiation sites that are activated with reduced efficiency throughout S phase and show much lower spatial limitation in the genome. Third, an increased density of lower frequency initiation sites has evolved in the larger, late-replicating chromosomes, potentially serving as a compensatory adaptation to ensure their efficient and timely replication within S phase.

Fork speed varies with replication timing

DNA combing has been used to measure replication fork speed in *Leishmania*,[55,57] but it could not ask if this was uniform across the genome. Here, we used DNAscent to map individual leftward and rightward moving DNA replication forks and determined their speed across the *L. major* genome by measuring individual fork lengths divided by BrdU pulse duration (Figure 3A). This analysis indicated a mean global fork velocity of 2.28 kb/min in asynchronously growing cells (Figure 3B), closely aligning with

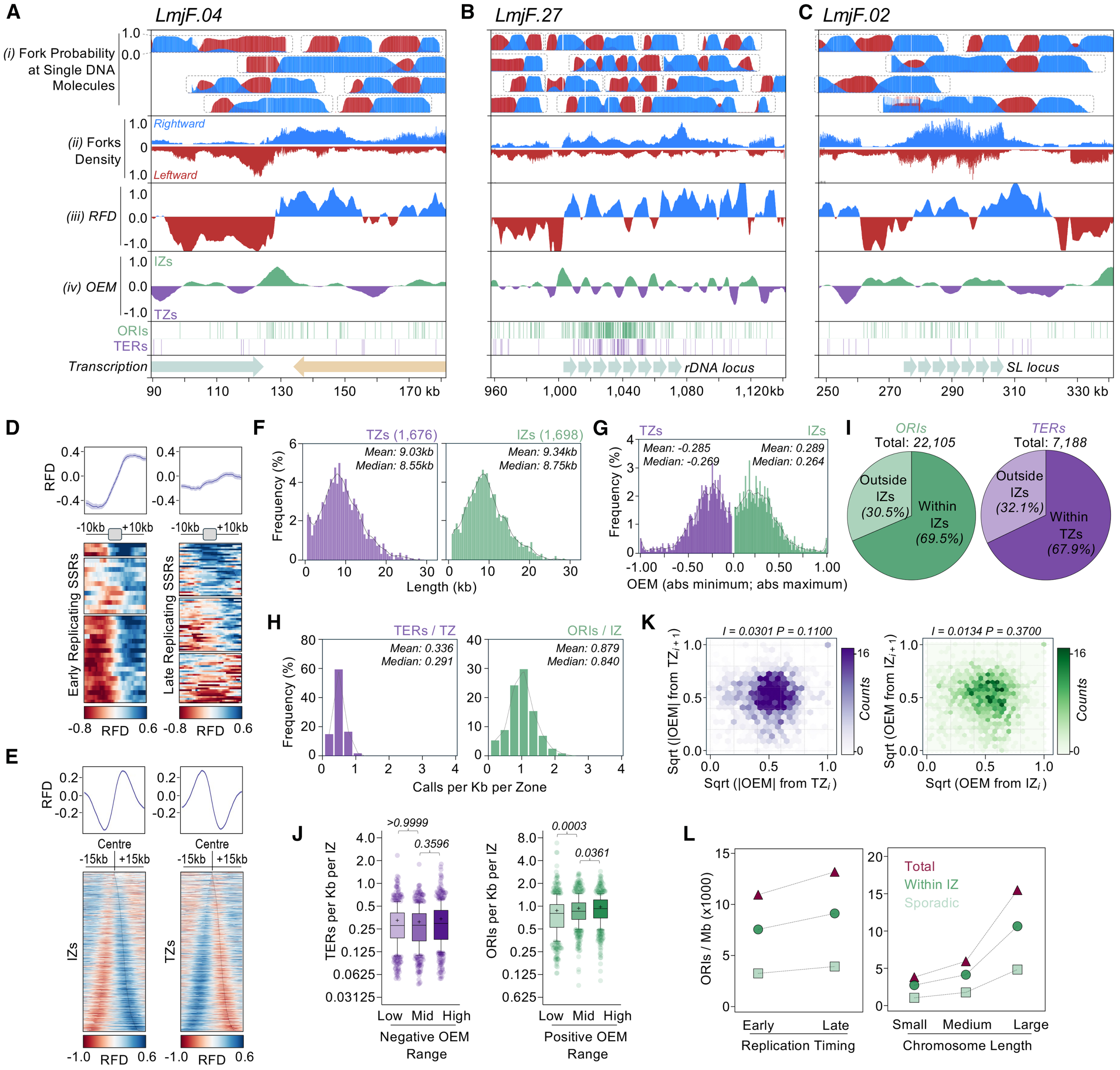

Figure 4. Analysis of DNA replication forks detected by DNAscent indicates *L. major* genome duplication relies on stochastic DNA replication initiation

Snapshots of (A) an early-replicating SSR, (B) the rDNA, and (C) SL loci. (i) Probability of leftward and rightward moving forks is shown for randomly selected Nanopore individual DNA molecules. (ii) Aggregated and normalized DNA replication fork density determined using forks called from single DNA molecules. (iii) Replication fork directionality (RFD) profiling, where positive and negative values indicate rightward and leftward fork movement, respectively. (iv) Origin efficiency metric (OEM) profiling, where positive and negative values indicate initiation zones (IZs) and termination zones (TZs), respectively. ORI and TER calls from single DNA molecules are shown as green and purple bars, respectively, below the OEM track.

(D) Summary plots (top) and colormaps (bottom) showing global RFD profile around all early-replicating SSRs (left) and all late-replicating SSRs (right) in NT cells.

(E) Summary plots (top) and colormaps (bottom) showing global RFD profile around all IZs (left) and all TZs (right) in NT cells.

(F) Frequency distribution of IZ and TZ lengths in NT cells.

(G) Frequency distribution of OEM maximum positive (IZ) and minimum negative (TZ) values in NT cells.

(H) Frequency distribution of ORI and TER density (calls per kb) within IZs and TZs, respectively, in NT cells.

(I) Genome-wide quantification of ORIs and TERs overlapping IZs and TZs, respectively, in NT cells.

(J) Boxplot of TER (left) and ORI (right) density within IZs and TZs, respectively, grouped according to OEM values. *p* values are indicated at the top. Statistical test, Kruskal-Wallis.

(legend continued on next page)

DNA combing estimates.[55,57] However, immediately following HU synchronization, a significant increase in global average fork speed to 2.54 kb/min was seen, subsequently declining to 2.51 kb/min at 15 min and to 2.25 kb/min at 60 min post release from HU arrest (Figure 3B). The explanation for this effect was revealed by comparing fork speed between early- and late-replicating genome compartments; among both unsynchronized and HU-synchronized cells, average fork speed was significantly higher in the former (Figure 3B). Moreover, average fork speed varied across chromosomes in a size-dependent manner, with earlier replicating small chromosomes showing greater average velocity than later replicating large chromosomes (Figure 3D). Notably, this size-dependent relationship was less pronounced 0 and 15 min after HU release, when most cells are in early S phase (Figure S1A) and DNA replication initiation is more focused on centromeric SSRs (Figure 2).

To explore potential factors influencing these spatial and temporal differences in fork speed, we quantified fork asymmetry and the occurrence of unidirectional forks, parameters frequently associated with replication stress and fork stalling. Fork asymmetry was assessed by calculating the ratio between the longer and shorter forks originating from the same initiation site (Figure 3A), while unidirectional forks were identified by molecules in which only a left- or right-moving fork was seen (Figure S4E). In asynchronous cells, we observed a median asymmetry ratio of 1.5 (Figure 3C), slightly exceeding previous values obtained by DNA combing,[55] likely reflecting DNAscent's capacity to examine more molecules. Immediately following release from HU synchronization, a subtle but significant increase in fork asymmetry was detected, but this returned to asynchronous levels by 60 min post HU release (Figure 3C). Notably, in either asynchronous or HU-synchronized cells, we detected no difference in fork asymmetry levels between early- and late-replicating genome compartments (Figure 3C) and found no significant correlation between fork asymmetry and chromosome length (Figure 3E). Similarly, though HU treatment transiently increased the length of unidirectional forks (Figure S4F), these were found at equivalent levels between genome compartments (Figure S4F) and across all chromosomes (Figure S4G).

Collectively, these observations suggest that replication forks traverse early-replicating compartments of the *L. major* genome faster than late-replicating ones. Importantly, these differences seem unrelated to localized variation in replication stress, suggesting that regulatory mechanisms govern DNA replication compartmentalization and timing in this parasite.

Origin efficiency metrics analysis provides a genome-wide mapping of DNA replication initiation and termination

To capitalize on DNAscent's analytical power, we generated genome-wide profiles of replication fork directionality (RFD) and origin efficiency metrics (OEMs)[66–68] by aggregating replication forks mapped by DNAscent, similar to analyses performed with FORK-seq in yeast.[9,15,65] RFD profiling provides a view of the predominant direction of replication forks at any given locus, with RFDs of −1.0 and +1.0 indicating 100% of left- or right-moving forks, respectively. OEM profiling indicates the extent of the upward or downward shifts in RFD profile, with positive and negative OEM values indicating zones of predominant fork divergence (initiation zones, IZs) and predominant convergence (termination zones, TZs), respectively. OEM values of +1.0 and −1.0 indicate, respectively, an IZ or a TZ predicted to be activated in 100% of replicating cells.

Representative examples of RFD and OEM profiles from unsynchronized cells are illustrated around the single early-replicating SSR on chromosome 4 of *L. major*, as previously predicted by MFA-seq and validated by DNAscent analysis (Figure 4A); as this SSR is flanked by converging PTUs, it is a site of transcription termination. Figure S4B provides representative examples of an early-replicating SSR that serves as a transcription start site for flanking diverging PTUs, as well as an early-replicating SSR that is flanked by tandemly oriented PTUs and is where transcription both terminates and starts. Consistent with the increased density of left- and right-moving forks surrounding early-replicating SSRs (Figure 1D), pronounced shifts in RFD were identified within each SSR. Furthermore, each of these early-replicating SSRs exhibited positive OEM values well over 0.5 (Figures 4A and S4B), corroborating their function as sites of frequent DNA replication initiation in unsynchronized *L. major* cells.

MFA-seq indicated early DNA replication initiation from the boundaries of the splice-leader (SL) and the ribosomal DNA (rDNA)[38] genes arrays, located at chromosomes 2 and 27, respectively. However, clear localization using short-read sequencing is challenging due to the repetitive nature of these loci. Nanopore sequencing coupled with DNAscent allowed the generation of RFD and OEM profiles for these genomic regions (Figures 4B and 4C). At the rDNA locus, a pronounced and localized upward shift in RFD was observed near the predicted transcription start site (Figure 4B). Notably, rightward fork movement, which is co-directional with transcription, exhibited oscillations across the locus, indicative of further DNA replication initiation events or fork pauses, similar to observations reported in yeast.[8] In agreement with the RFD, five regions with smaller OEM values and abundant ORIs were predicted within the rDNA locus, perhaps suggesting multiple IZs across the repeats. Though a less pronounced upward shift in RFD, as well as a lower OEM, was detected at the predicted transcription start site of the SL locus (Figure 4C), there was less evidence of further IZs or putative pauses across the SL repeats, suggesting that most DNA replication initiation occurred at the promoter. Nevertheless, these findings corroborate the rDNA and SL loci as prominent DNA replication initiation loci for each chromosome.

(K) Hexagonal bins density plot comparing termination efficiency between a given TZ (TZ_i) and its neighbor (TZ_{i+1}) or initiation efficiency between a given IZ (IZ_i) and its neighbor (IZ_{i+1}). Absolute OEM values (|OEM|) were used for the TZ analysis. Square root transformation (Sqrt) was applied to both *x* and *y* axis for visualization purposes. *I* and *P*, spatial autocorrelation index and *p* value, respectively, as determined by Moran's I autocorrelation test.

(L) Distribution of ORI types between early- and late-replicating genome compartments (as determined by MFA-seq), and between chromosomes grouped according to their size (small, 0.27–0.62 Mb; medium, 0.63–0.84 Mb; large, 0.91–2.68 Mb).

To examine RFD and OEM profiles at a genome-wide level, we first performed metaplot analysis around SSRs. RFD switching around early-replicating SSRs was significantly more pronounced than at late-replicating SSRs (Figure 4D). Furthermore, when comparing RFD switching between SSRs grouped according to the arrangement of flanking PTUs, we observed that RFD switching was less pronounced at convergent SSRs and more pronounced at divergent and head-to-tail SSRs, which are more frequently the sites of early DNA replication initiation (Figure S4C) and are reflected in relative OEM values (Figure S4D). Taken together, these data suggest that early replication timing, as initially predicted by MFA-seq,[38] is a feature of *Leishmania* centromeric SSRs, rather than simply an association with sites of transcription termination or initiation, which occur at all SSRs.

The aforementioned data are focused on the SSRs, where ORC binding is seen in *T. brucei*.[27,33] However, large numbers of regions of upward and downward RFD switching are seen beyond the SSRs (Figures 4A–4C and S4B), consistent with previously undetected, genome-wide initiation and termination events (Figure 2). Indeed, OEM analysis predicted 1,698 IZs and 1,676 TZs, with average lengths of 9.34 and 9.06 kb, respectively (Figure 4F). This number of IZs equates to DNA replication initiation at ~18.8 kb intervals across the genome at the population level, consistent with a median inter-ORI distance of 20.7–22.3 kb observed at the single-molecule level (Figure S3C). Metaplot analysis confirmed the expected converging and diverging RFD profiles around IZs and TZs, respectively, as well as heterogeneity in the location of RFDs across fixed-size genome fragments (Figure 4E). To determine the overall efficiency of these zones, we examined the range of OEM values for both IZs and TZs (Figure 4G). Only a small fraction (~0.5%) of IZs and TZs had an OEM close to +1.0 or −1.0, respectively, indicating that highly efficient zones in which replication initiation or termination occurs in a spatially and temporally synchronized manner in replicating cells are rare. Instead, the mean OEM values of +0.289 and −0.285 for IZs and TZs, respectively, indicate the replication program in *Leishmania* relies on potentially stochastic and low-efficiency zones of initiation and termination that are, on average, used simultaneously in only 28% of replicating cells within the population.

We also examined the density of initiation sites overlapping IZs and found an average of 0.879 ORIs per kb within these regions (Figure 4H), with increased ORI density in higher efficiency IZs (Figure 4J). At the TZs, we found an average density of 0.336 termination sites per kb (Figure 4H) and no correlation with TZ efficiency (Figure 4J). By examining the intersection of ORIs and terminations with IZs and TZs, respectively, we observed that approximately 30% of ORIs and 32% of termination sites were located outside TZs and IZs, respectively (Figure 4I). This proportion substantially exceeds the 9% of origins outside IZs predicted in yeast by comparable analysis.[9] Instead, our observations align more closely with patterns seen in human cells, where the majority of DNAscent-predicted initiation sites occur outside of IZs mapped with Okazaki fragments sequencing (OK-seq).[17] Consequently, our data suggest a replication model for *L. major* reminiscent of that proposed for human cells,[69] comprising a small number of "master" IZs and a broader landscape of dispersed, "sporadic" initiation events (Figure 2H). Calculating the density of sporadic ORIs in early- or late-replicating compartments of the chromosomes, as well as in chromosomes categorized by size, showed them to be found more frequently in late-replicating regions (Figure 4L), consistent with a higher density of DNAscent-predicted ORIs in the larger, later replicating chromosomes of unsynchronized cells (Figure 2G). Finally, we asked whether initiation and termination efficiency in neighboring IZs and TZs show correlation, which would indicate that proximity influences activity (Figure 4K). However, by comparing the minimum OEM values from adjacent TZs and the maximum OEM values from adjacent IZs, we did not find any significant correlation.

Given the wide distribution of ORIs (Figure 2B and 2C), the low correlation in ORI usage between adjacent genomic regions (Figures 2E and 4K), the low average efficiency of IZs (Figure 4G), and the high proportion of sporadic initiation events (Figure 4I), we propose that a large component of the genome duplication program of *L. major* relies on stochastic, non-centromeric DNA replication initiation events.

IZs and TZs identified by DNAscent are regions of localized variation in sequence content and chromatin accessibility

In an attempt to identify features associated with DNAscent-predicted DNA replication events, we performed metaplot analyses of all IZs and TZs identified by OEM and examined their average sequence content (Figure 5A). Although no conserved sequences were found, IZs were notable as regions of increased AT content, whereas TZs were found in GC-enriched loci. In addition, analysis of G4-seq data[60] showed evidence for mild G4 formation on both DNA strands at IZs, whereas moderate G4 under-representation was seen at TZs. MEME motif analysis indicated that G4s within IZs are predominantly of the relatively unstable $G_{2+}L_{1-12}$ class (Figure S5A), mainly detectable after chemical stabilization.[60] The mapping of MNase-seq[57] data showed that IZs are found in areas of lower chromatin occupancy, while TZs displayed more compact chromatin. Importantly, the chromatin differences between IZs and TZs could not be attributed to variations in poly(dG:dC) sequence representation (Figure S5B), which are associated with nucleosome exclusion in other eukaryotes,[70] though IZs exhibit a mild enrichment of G_{3-5} tracks, but only when overlapping the G4s within these regions (Figure S5C).

Each of the aforementioned genomic features of IZs positively correlates with IZ efficiency (Figure 5B); when IZs were separated into four different groups according to their average OEM, the extent of AT enrichment, depletion of GC, G4 level, and chromatin accessibility increased from low to high. We therefore compared OEMs in early- and late-replicating genome compartments (Figure 5C), as well as in three different groupings of chromosome size (Figure 5D). In neither analysis were significant differences found in OEM mean or range, suggesting no differential efficiency of IZs dependent on overall replication timing or chromosome size. Nonetheless, late-replicating areas of the genome were more enriched in IZs with lower chromatin occupancy (Figure 5C) and, furthermore, IZs showed increasing AT levels, lower GC levels, and increasing chromatin accessibility as chromosome size increased (Figure 5D). Moreover, metaplot

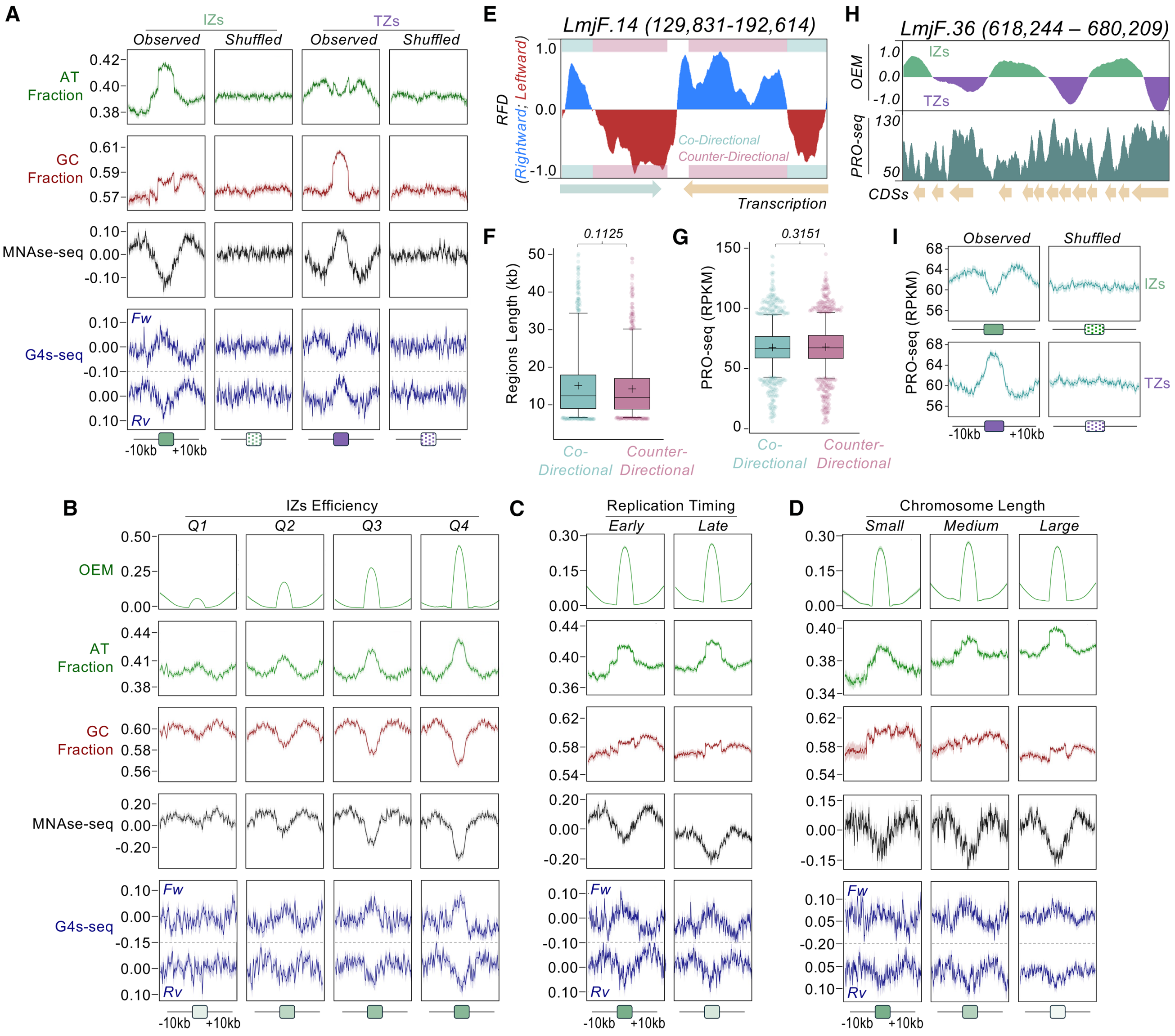

Figure 5. DNA sequence content and chromatin accessibility are related to DNA replication initiation efficiency

(A) Metaplots showing global AT and GC content, MNase-seq, and G4-seq profiles around IZs and TZs from NT cells. As controls, profiles were generated after the same regions were randomly redistributed in the genome (shuffled).

(B) Same analysis as in (A), but comparing IZs grouped into quartiles (Q1, less efficient; Q4, most efficient), defined according to the maximum positive OEM value from each region.

(C) Same analysis as in (A) but comparing IZs from early- and late-replicating genome compartments, as determined by MFA-seq.

(D) Same analysis as in (A) but comparing IZs from distinct chromosomes grouped according to their length (small, 0.27–0.62 Mb; medium, 0.63–0.84 Mb; large, 0.91–2.68 Mb).

(E) Representative snapshot showing RFD profile at the indicated segment of chromosome 14. Arrows at the bottom indicate transcription direction. Co-directional (light pink), RFD and transcription in the same direction. Counter-directional (light teal), RFD and transcription in opposite directions.

(F and G) Comparing lengths (F) and levels of nascent RNA (PRO-seq) (G), respectively, from co-directional and counter-directional genome compartments. Horizontal line and cross, median and mean, respectively. p values are indicated at the top. Statistical test, Kruskal-Wallis.

(H) Representative snapshot comparing OEM profile and PRO-seq data in the indicated segment of chromosome 36.

(I) Metaplots showing global levels of nascent RNA around IZs and TZs. As controls, profiles were also generated after the same regions were randomly redistributed in the genome (shuffled).

analysis revealed that higher ORI usage positively correlated with increased OEM values, greater AT enrichment, GC depletion, and elevated G4 levels (Figure S5D). These data suggest that despite increased ORI usage in larger, later replicating chromosomes, the average efficiency of IZs is similar across replication timing compartments.

IZs are regions with reduced levels of nascent RNA, whereas TZs are associated with increased levels of nascent RNA

The near-ubiquitous use of multigenic transcription potentially represents a particular problem for genome duplication in kinetoplastids: the need for RNA polymerase (Pol) II to continuously traverse long genomic distances may result in regions of pronounced collisions with the replisome. In both *T. brucei*[33] and *L. major* (here and[38,39]), MFA-seq and now DNAscent indicate that DNA replication initiation in early S phase is most pronounced around the transcription start or stop sites of select PTUs, but no work has asked if and how transcription and replication might intersect in *Leishmania*. To address this question, we intersected the RFD profiles derived from DNAscent with annotated transcription direction and thereby identified genomic segments where DNA replication and transcription travel in the same direction (co-directional), as well as segments where DNA replication and transcription are in opposition (counter-directional; Figure 5E). We did not observe any significant difference between the sizes of co-directional and counter-directional regions (Figure 5F), indicating a lack of selection for or against one or the other arrangement. Furthermore, we took advantage of recently published PRO-seq analysis[71] and observed that levels of nascent RNA transcripts are similar in co-directional and counter-directional segments (Figure 5G). Altogether, these data suggest that the genome organization in this parasite did not evolve to favor co-directional movement of the DNA replisome and transcription machinery, and DNA replication direction does not seem to influence transcription initiation efficiency. However, visual inspection showed that PRO-seq levels were not uniform across a PTU, and there appeared to be a potential correlation between the areas of decreased and increased PRO-seq relative to predicted IZs and TZs, respectively (Figure 5H). To test this prediction, we performed metaplot analysis of PRO-seq data centered on all predicted IZs or TZs and 20 kb of the surrounding sequence (Figure 5I). This analysis revealed a clear decrease in PRO-seq signal within the IZs and an increase within the TZs. Taken together, these data suggest that transcription and DNA replication intersect within PTUs, with sites of reduced RNA Pol II transcript level corresponding with stochastic DNA replication initiation, and the inverse correlation between areas of increased transcripts and stochastic DNA replication termination.

Limited correlation between initiation sites predicted by DNAscent and SNS-seq

Next, we asked if the DNA replication initiation sites predicted by DNAscent correlate with initiation sites mapped by SNS-seq.[57] By allowing a maximum distance of 1 kb between calls midpoints, we found that only ~24% of ORIs identified by DNAscent overlapped with SNS-seq signals (Figure 6A). Furthermore, metaplot analysis revealed that DNAscent-derived ORIs frequently exhibited positional offsets either upstream or downstream relative to initiation sites detected by SNS-seq (Figure 6B), with only ~11% showing clear overlap. Reciprocal analysis showed that ~56% of initiation sites identified by SNS-seq corresponded with ORIs mapped by DNAscent (Figure 6A). In this case, metaplot analysis revealed clearer overlap of SNS-seq signal and DNAscent-detected ORIs, but still in a minority of cases (~24%; Figure 6C). One explanation for the poor overlap between these two datasets is that DNAscent, operating at single-molecule resolution, has greater sensitivity, enabling detection of potentially every initiation site, whereas SNS-seq, a population-based approach, preferentially captures higher abundance events. Supporting this notion, DNAscent-predicted ORIs overlapping SNS-seq sites exhibited a mild but significantly increased usage density compared to non-overlapping ones (Figure 6D), whereas no significant difference was seen between SNS-seq signal levels at sites overlapping or not overlapping DNAscent-predicted ORIs (Figure 6E).

To ask if SNS-seq mapping correlates with genome features similar to those seen at DNAscent ORIs (as described earlier), we performed metaplot analyses of all SNS-seq initiation sites, examining sequence composition and chromatin (Figure 6F). There were similarities in that SNS-seq signal was seen in regions of lower chromatin occupancy and G4 enrichment, as previously reported,[57] as well as increased AT content and lowered GC content. However, AT content increase and lowered chromatin occupancy were more marked at loci where SNS-seq signal and DNAscent ORIs overlapped, while reduced GC content was not seen at SNS-seq loci that did not correlate with DNAscent, and G4 enrichment was notably more localized at SNS-seq loci than at DNAscent ORIs (Figures 6F and 5A). Moreover, RNA:DNA hybrids (R-loops), which localize to inter-CDS regions throughout PTUs in both *L. major*[72] and *T. brucei*,[73] show considerable correlation with SNS-seq signal, an association that is substantially less marked at initiation loci predicted by both SNS-seq and DNAscent (Figure 6F). Indeed, as we recently reported, in asynchronous *L. major* cells, SNS-seq density increases as chromosome size decreases,[64] which is the opposite correlation to that we detect here for DNAscent ORIs (Figure 2G); consistent with this, the direct comparison between average SNS-seq signal and DNAscent ORI usage per chromosome revealed a significant anti-correlation (Figure 6G). Taken together, these analyses may suggest that DNAscent and SNS-seq detect different processes, or the greater sensitivity of DNAscent can detect a larger and more widely distributed number of initiation events than population-level SNS-seq mapping.

DNA replication initiation efficiency and timing correlate with genome variation

To ask if DNA replication influences genome variability in *L. major*, we performed metaplot analysis to examine the density of SNPs that form after growth in culture around IZs and TZs. Consistent with observations in human cells showing that DNA replication initiation leads to mutagenesis,[74] we observed increased accumulation and mild depletion of SNPs around DNAscent IZs and TZs, respectively (Figure 7A). Moreover, the accumulation of SNPs within IZs correlates with their efficiency; when IZs were separated into four groups according to average OEM, SNP enrichment increased from low to high OEM (Figure 7B).

Next, we asked if the temporal organization of DNA replication across the *L. major* genome correlates with DNA replication initiation efficiency and mutation patterns. Using our MFA-seq-based DNA replication timing data,[38,39] we grouped the genome into four genome compartments (Figure 7C): early-replicating centromeric SSRs, late-replicating SSRs, subtelomeres (which exhibit persistent replication throughout the cell cycle[39]), and

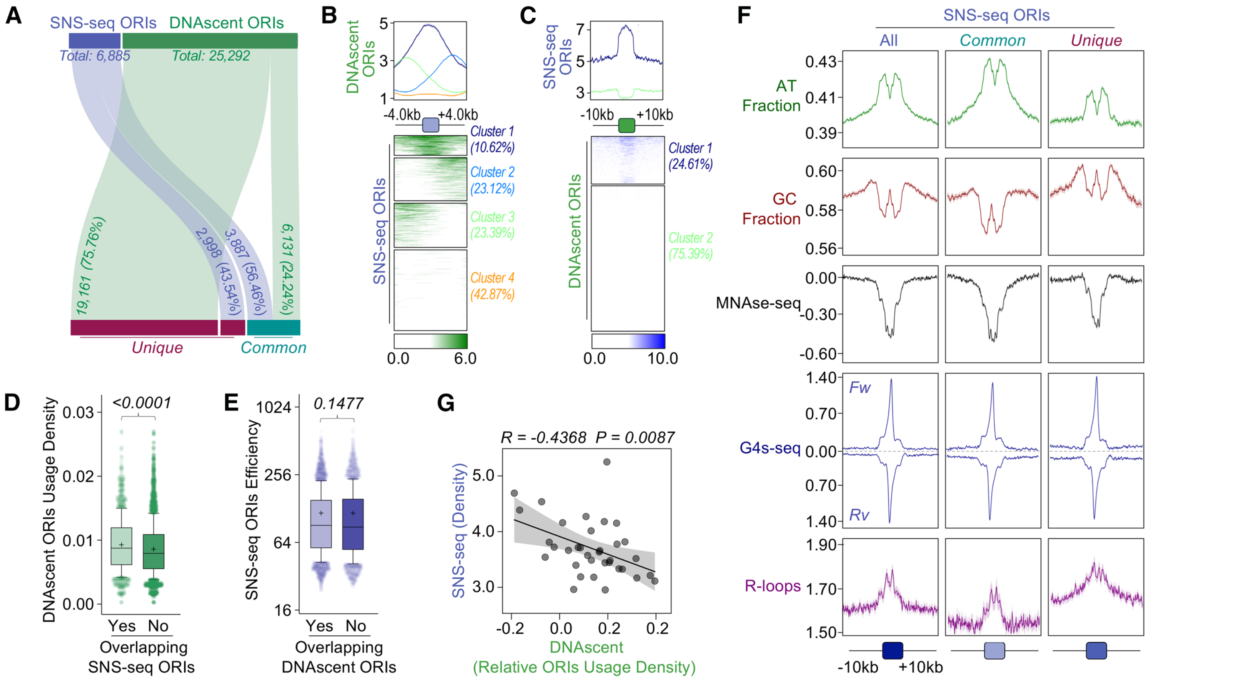

Figure 6. Comparison of DNAscent and SNS-seq

(A) Alluvial plot showing the quantification of overlapping or distinct DNAscent and SNS-seq ORIs in NT cells.

(B) Summary plots (top) and colormaps (bottom) showing DNAscent ORI density around all SNS-seq ORIs in NT cells.

(C) Summary plots (top) and colormaps (bottom) showing SNS-seq ORI density around all DNAscent ORIs in NT cells.

(D and E) Comparison of DNAscent ORI usage (D) and SNS-seq ORI efficiency (E), respectively, based on the overlap with each other. *p* values are indicated at the top. Statistical test, Mann-Whitney.

(F) Metaplots showing global AT and GC content, MNase-seq, G4-seq, and DRIP-seq profiles around SNS-seq ORIs in NT cells.

(G) Simple linear regression analysis between average SNS-seq ORI density and average DNAscent ORI usage at each chromosome. Shaded areas represent 95% confidence intervals. *R* and *p* values are indicated at the top.

CDSs (representing PTUs). Comparing MFA-seq signal averages between these compartments confirmed the expected DNA replication timing differences, with centromeric SSRs being the earliest replicating (Figure 7D). This timing correlated with OEM profiles (Figure 7E) and with predicted ORI density (Figure 7F), with early-replicating SSRs having the highest average OEM and being IZs with higher ORI density compared to the three other compartments. A more detailed analysis using metaplots revealed that ~53% of early-replicating SSRs (Figure 7G, clusters 1 and 2) closely overlapped with IZs exhibiting an average OEM of 0.5–0.6. The remaining early-replicating SSRs (Figure 7G, clusters 3 and 4) either partially overlapped or were flanked by IZs with OEMs of ~0.2. In contrast, ~75% of late-replicating SSRs (Figure 7H, clusters 1–4) coincided with IZs with average OEMs of 0.2–0.3, while 25% did not overlap with IZs (Figure 7H, cluster 5). Comparing OEMs between SSRs grouped according to flanking PTU transcription direction showed increased average OEM correlated with the proportion of early-replicating SSRs in each group (Figure S4D). ~74% of subtelomeric regions contained IZs with OEMs <0.3 (Figure 7I, clusters 1–3), and 25% displayed negligible IZ efficiency (Figure 7I, cluster 4). Finally, OEM profiling revealed asymmetric DNA replication activity around CDSs, with IZs and TZs predominantly localized to one side or the other of approximately two-thirds of the sequences (Figure 7J, clusters 1 and 2); notably, the average OEM across the CDS was the lowest compared with the other genome compartments. Collectively, these data suggest that early DNA replication from centromeric SSRs is mediated by the most efficient and ORI-dense IZs.

At each of these compartments, we then assessed mutation levels by comparing the density of SNPs that formed after growth in culture (Figure 7K). The highest SNP accumulation was seen at early-replicating SSRs, consistent with these being sites of the most frequent DNA replication initiation. At both late-replicating SSRs and subtelomeres, some SNP accumulation was also seen, but at lower levels, reflecting the lower OEMs and greater proportions that appeared not to be IZs. Finally, modest but detectably asymmetric accumulation of SNPs was seen around CDSs, mirroring the OEM profile. Taken together, these data indicate that DNAscent-predicted IZs are loci of increased mutagenesis, and, moreover, the level of such mutagenesis reflects the measured efficiency of DNA replication initiation and timing.

DISCUSSION

Analysis of nuclear DNA replication in the ubiquitous human and animal pathogen *Leishmania* has, to date, relied on population-level next-generation sequencing approaches.[38,39,57] Here, we

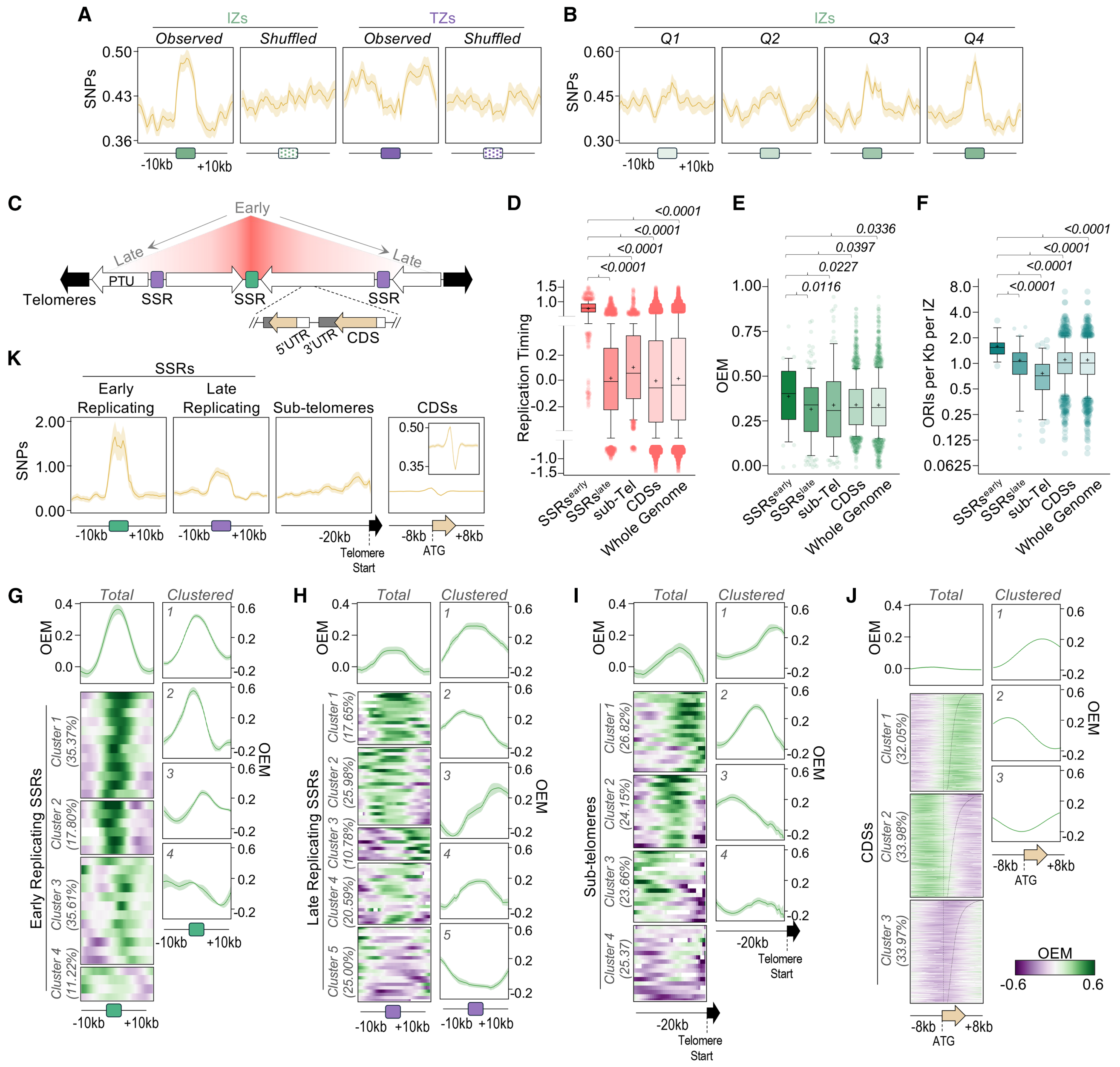

Figure 7. Mutation levels at zones of DNA replication initiation correlate with initiation efficiency and replication timing

(A) Metaplots showing global SNP density around IZs and TZs from NT cells. As controls, profiles were also generated after the same regions were randomly redistributed in the genome (shuffled).

(B) Same analysis as in (A), but comparing IZs grouped into quartiles (Q1, less efficient; Q4, most efficient) defined according to the maximum positive OEM value from each region.

(C) Schematic of general genome organization in *L. major*. PTU, polycistronic transcription unit; SSRs, regions where PTU transcription starts and/or ends; SSRs overlapping the centromere in each chromosome are the earliest replicated, as determined by MFA-seq (salmon).

(D–F) Comparison of replication timing (D), maximum positive OEM values (E), and density of ORI calls (F) within IZs, respectively, between the indicated genome compartments. Horizontal line and cross, median and mean, respectively. *p* values are indicated at the top. Statistical test, Kruskal-Wallis.

(G–J) Summary plots (top) and colormaps (bottom) showing global OEM profiles from NT cells around all early-replicating SSRs (G), late-replicating SSRs (H), subtelomeres (I), and CDSs (J), respectively. Each row on the colormaps represents an individual region belonging to that genome compartment. For each genome compartment, *K-means* clustering was used to group regions according to their OEM profile. Summary plots of the OEM profile from each cluster are shown to the right of the colormaps. The proportion of regions in each cluster is shown to the left of the colormaps.

(K) Metaplots showing global SNP density around the indicated genome compartments in NT cells.

have made use of the capacity of Nanopore sequencing to detect the nucleoside analog BrdU in long sequence reads, first described by Muller et al.,[8] to perform single-molecule analysis of DNA replication in *L. major* and understand how this eukaryotic parasite copies its genome. Our work not only confirms the unprecedented use of just a single locus for high-efficiency DNA replication initiation in each *L. major* chromosome but also reveals thousands of widespread initiation events that escaped detection by all previous approaches. Furthermore, we show that the distribution of these abundant initiation events relates to the unusual chromosome size-dependent timing of *L. major* DNA replication. Finally, we show that all predicted sites of DNA replication initiation are marked by localized patterns of base content, chromatin organization, transcription, and mutation, revealing that the program for DNA replication adopted by *Leishmania* has shaped the evolution of the parasite's genome.

The first attempt to map the origins in *Leishmania* species relied on MFA-seq, an approach that in *S. cerevisiae* and relatives shows considerable consistency with locations of conserved origins,[3,75–77] and in *T. brucei*, clearly correlates with the binding locations of one subunit of ORC.[33,34] In both cases, and in accordance with the eukaryotic DNA replication canon, multiple origins were detected per chromosome. In *Leishmania*, however, MFA-seq analysis of two species, *L. major* and *L. mexicana*, was only able to detect a single putative origin of replication in each chromosome.[38] This unprecedented observation was met with caution, primarily because one origin per chromosome, although in theory enough to replicate many of the smaller chromosomes during S phase, is undoubtedly insufficient to replicate the larger parasite chromosomes completely.[28,32,38] Indeed, we hypothesized that other origins must be present but are less efficient or stochastic (and thus eluded MFA-seq detection). Indeed, further refinement of our MFA-seq approach suggested that subtelomeric replication may occur outside S phase and is dependent on components of the 9-1-1 DNA checkpoint clamp (involved in DNA repair).[39] Studies using other methodologies have challenged the MFA-seq findings,[31,55] with the only other genome-wide analysis, SNS-seq, predicting ∼5,000–6,000 putative origins and limited correspondence to MFA-seq data.[57] Thus, a complete understanding of how *L. major* replicates its genome remained elusive. DNAscent[8,61] is a methodology that detects DNA replication in single DNA molecules and allows the prediction of fork direction, leading to predictions of initiation and termination loci across the genome. DNAscent has a number of advantages over previous approaches applied to *Leishmania*: it is much more sensitive than MFA-seq, in that it examines long single DNA molecules, provides for genome-wide analysis, which DNA combing approaches used so far in *Leishmania* do not, and relies only on the detection of BrdU incorporated into sequenced DNA, thus circumventing the need for processing and enrichment to detect replicating DNA molecules. We suggest that the use of DNAscent more accurately predicts sites of DNA replication initiation in *L. major* than any previous analysis, based on two observations. First, DNAscent confirms and extends the findings of population-level MFA-seq mapping, cross-validating the two approaches. Second, DNAscent predicts more widespread sites of initiation than MFA-seq or any other approach used to date. Despite these advances, two different scenarios might explain what is now known about DNA replication programming in *L. major* (Figure 2H).

One scenario is that DNA replication of each *L. major* chromosome relies on a single constitutive, ORC-defined origin supplemented by much more widespread stochastic initiation events (ORIs). Constitutive initiation at a single locus at the onset of S phase is consistent with several aspects of DNAscent mapping. First, the most prominent site of BrdU signal density in Nanopore reads, allied to DNAscent analysis of the pattern of BrdU accumulation across the genome, shows that the only loci where signal is consistently enriched in unsynchronized *L. major* promastigote cells correspond to a single SSR in each chromosome, overlapping MFA-seq mapping (Figures 1B, 1C, S1C, S2H, and S2G). In addition, HU-mediated G1/S synchronization of the cells, followed by release and synchronous progression across early S phase, is consistent with coordinated bidirectional replication fork movement from only these loci and with striking consistency of movement between chromosomes (Figures 1D and S4A). Finally, RFD predictions suggest that DNA replication initiates from relatively discrete locations within most of these SSRs (Figures 4A , 4D, S4B, and S4C). No study has yet described ORC genomic localization in *L. major*, and so we cannot say if these early-replicating SSRs are true origins or are instead loci at which wider initiation sites are concentrated (as described further). Nonetheless, a range of data support the hypothesis that these early-replicating SSRs are discrete, ORC-defined origins. As we have noted before,[38] a significant fraction of the early-replicating SSRs in *L. major* are syntenic with ORC-bound origins in *T. brucei*. The abrupt RFD shifts seen by DNAscent within most SSRs appear more comparable to the RFD shifts seen at sequence-defined origins in *S. cerevisiae*[9,67,68] than the more diffuse RFD shifts seen in human cells (either because of greater heterogeneity of origin usage or clusters of origins).[67,69] These SSRs appear also to be the locations of *L. major* centromeres,[54] which are also found in single copy in each chromosome of *T. brucei* and are coincident with the earliest replicating orgins,[33] even when they reside in the transcriptionally silent, late-replicating subtelomeres.[35] Thus, the earliest or most efficient DNA replication activation events in S phase of these two related parasites appear to rely on so-far unexplored links with centromere function; that this activity would involve ORC localization in *Leishmania* is consistent with BioID analysis indicating proximity of the kinetochore and ORC in *L. mexicana.*[78] Where *T. brucei* and *L. major* appear to differ in this model is in how they program DNA replication across the genome and cell cycle beyond such centromere-focused origin activity. In *T. brucei*, every SSR binds ORC and around 25% act as origins that can be detected by MFA-seq[33]; in contrast, none of MFA-seq,[38,39] SNS-seq,[57] or DNAscent (this study) provide evidence that non-centromeric SSRs are equivalent, ubiquitous sites of DNA replication initiation in *L. major*.

Theoretical predictions suggest that the relatively small number of ORC-localized origins detected at SSRs by MFA-seq in each *T. brucei* chromosome is around the minimum needed to complete copying of each chromosome in S phase and does not provide an excess of potential back-up origins, as seen in yeast, for example.[79] Here, DNAscent indicates that *Leishmania*

uses a different strategy to complete the replication of each of its chromosomes: high-efficiency DNA replication initiation at the centromeric SSRs early in S phase is supplemented with the abundant use of less-efficient ORIs, which are distributed across chromosomes and do not specifically localize to SSRs. The total number of such stochastic *L. major* ORIs is hard to determine from the available data, as such measures will depend on sequence depth. Nonetheless, as DNAscent examines DNA replication patterns on single molecules, it is somewhat comparable to single-molecule combing, which previously predicted inter-origin distances in *L. major* of ~70 kb[58] and ~195 kb[56]; here, DNAscent predicts an inter-origin distance of ~20 kb, which is likely to be close to the minimum detectable by fiber analysis,[57] and suggests the use of substantially larger numbers of ORIs than any previous study has suggested.[38,39,55,57] Most likely, the combination of the sheer number of such ORIs, allied to flexibility in their localization in the genome, precludes their detection by MFA-seq. In this regard, SNS-seq has also previously predicted DNA replication initiation sites in the *L. major* genome that were not seen by MFA-seq,[57] but we find limited overlap between DNAscent and SNS-seq data (Figure 6), which contrasts with the good correspondence between SNS-seq and Nanopore-BrdU mapping in *P. falciparum* cells undergoing schizogony.[23] Further work will be needed to resolve the dichotomy between SNS-seq and DNAscent mapping (see limitations of the study), but one intriguing possibility is that *L. major* employs two distinct forms of DNA replication initiation in addition to the high-efficiency centromeric SSR-focused activity. For instance, while all our data suggest that DNAscent detects truly stochastic events that show no clear evidence for spatial limitation, SNS-seq predicts DNA replication initiation, potentially driven by G4 structures or R-loops (Figure 6), that is mainly localized to inter-CDS regions in the *L. major* PTUs. Why these events would evade detection by DNAscent is unclear, and it is also not clear why DNAscent mapping readily detects activity at centromeric SSRs, while such correspondence is unclear with SNS-seq.[57] Irrespectively, the abundance of stochastic ORIs in *Leishmania* may be truly unusual among single-celled eukaryotes. In *S. cerevisiae*, only around 10%–20% of origins mapped by BrdU-Nanopore sequencing cannot be aligned with known origins,[8,9] whereas 80% of DNAscent-predicted initiation events in human cells do not match a range of origin prediction approaches.[17] Thus, it is possible that *Leishmania*'s abundant use of stochastic DNA replication initiation may have parallels with at least some metazoans.[10]

A distinct scenario from the above is that all DNA replication initiation in *L. major* is stochastic, and thus, there is no mechanistic distinction between initiation at the single centromeric SSR in each chromosome and at the more abundant DNAscent ORIs (or indeed at SNS-seq signals). In this scenario, high-efficiency initiation at the centromere is not due to a narrow focus on the SSR but concentration of locus-unspecific stochastic initiation events around the centromeric SSR, with loosening of such spatial localization at more distal parts of the chromosomes (Figure 2H). How stochastic initiation events might be spatially concentrated is unclear, but this scenario would explain why OEM measurements do not suggest activation at the centromeric SSR in every cell (Figure 4) and may explain the distribution of predicted ORIs around this locus before and after HU synchronization (Figure 2).

From the available data on DNA replication, we can only speculate on the nature of the abundant, stochastic ORIs in *L. major*. It is possible these ORIs are also designated by ORC binding, and therefore *L. major* has a previously unanticipated abundance of "true" origins. However, ORC designation of such widely dispersed origins may be problematic for *Leishmania*, since in other eukaryotes, ORC associates with DNA and recruits the MCM helicase in G1. As most of the DNAscent-predicted ORIs are spread throughout the PTUs of *L. major*, they might be predicted to present a much greater impediment to transcription than in other eukaryotes, given the ubiquitous use of polycistronic transcription. It is possible that ORC might be loaded, perhaps at the single early-replicating SSR, in G1, but is loosely bound and mobile, and so moves throughout the genome until the activation of DNA replication in S phase, which could then occur highly flexibly, wherever ORC is found. Alternatively, it has been suggested by Lombraña et al. [57] that ORC recruitment of MCM might lead to a stable pre-RC complex only at the centromeric SSRs, with MCM in other locations free to move away from ORC and lead to initiation. Both suggestions are consistent with the lack of any conserved sequence features of the ORIs, and the shifts in localized base and chromatin content, as well as nascent transcript levels, we observe at the initiation zones, might reflect loci where the replication machinery lingers in the genome. Finally, is it possible that stochastic initiation events are ORC independent? Such activator-independent initiation of DNA replication has been described in eukaryotes but is normally only readily detected as a "back-up" reaction after mutation of the activator-origin machinery or *in vitro*. [80–84]

How *Leishmania* might program the use of localized, high-efficiency DNA replication initiation alongside dispersed, lower efficiency initiation in each chromosome will require clarification about their mechanistic overlap. Nonetheless, the difference in fork speed we detect in early- versus late-replicating genome compartments (Figure 3B), as well as the greater average fork speed in early-replicating small chromosomes compared to late-replicating large chromosomes that are enriched in stochastic ORIs (Figure 3D), may hint at different forms of DNA replication initiation. Alternatively, differences in fork speed may relate to unexplored differences between early- and late-replicating genome compartments, such as chromatin or subnuclear organization. We have previously reported subtelomeric DNA replication in *L. major* that appears not to be limited to S phase and is dependent on Rad9,[39] but it is unlikely that this reaction accounts for all stochastic DNA replication reactions, since DNAscent-predicted ORIs are not limited to this genome compartment. A further question raised by this study is why *Leishmania* has evolved such a bimodal DNA replication program. An explanation may lie in directing patterns of genome change. Here, we show that SNPs accumulate at all zones of DNA replication initiation, but these are most pronounced at the earliest replicating locus in each chromosome, confirming and extending our previous analyses.[39,40] The same effect has been observed at human origins, with patterns of mutation differing in "core" and cell-type-specific origins.[74] *Leishmania* may then have evolved to spread such initiation-induced

CellPress
OPEN ACCESS

mutagenesis across the genome, rather than focusing it at a small number of sites, as this could promote adaptation. Intriguingly, recent work using MFA-seq mapping has revealed distinct levels of predicted origins in the highly transcribed core and largely untranscribed subtelomere compartments of the *T. brucei* genome, with the latter compartment having MFA-seq peaks and displaying notably greater instability.[35] No work has applied DNAscent in *T. brucei*, and so it remains unclear if subtelomere DNA replication may rely on similar stochastic ORIs that are found genome-wide *in L. major*. Nonetheless, the data we present here provide a mechanistic link between DNA replication programming and genome plasticity in *Leishmania*, which may have parallels with other kinetoplastids.

Limitations of the study

The key limitation of this work, which is shared with previous SNS-seq mapping,[57] is the lack of evidence that the predicted initiation events are truly origins of DNA replication. Though DNAscent predictions correlate with previous population-level MFA-seq showing DNA replication initiation at the single centromeric SSR in each chromosome,[38,39] the wider stochastic ORIs have not been seen previously and therefore remain predictions that require testing, such as by correlation with replication machinery binding or demonstration that their localization or activity can be perturbed genetically.

RESOURCE AVAILABILITY

Lead contact

Requests for further information and resources should be directed to and will be fulfilled by the lead contact, Richard McCulloch (richard.mcculloch@glasgow.ac.uk).

Materials availability

No unique reagents were used.

Data and code availability

ONT sequences used for DNAscent analysis are available at the European Nucleotide Archive under accession number PRJEB82099; no new code was used.

ACKNOWLEDGMENTS

We thank Mike Boemo for the helpful discussions about establishing DNAscent in *Leishmania* and all current and previous members of the McCulloch lab for input. This work was supported by the Wellcome Trust (224501/Z/21/Z), the BBSRC (BB/N016165/1, BB/R017166/1, and BB/W001101/1), the MRC (MR/S019472/1), and the European Union's Horizon 2020 research and innovation program under the Marie Sklodowska-Curie grant agreement no. 750259 (Individual Fellowship, RECREPEMLE). The Wellcome Center for Integrative Parasitology was supported by core funding from the Wellcome Trust (104111). Parts of Figure 1A were generated using BioRender.

AUTHOR CONTRIBUTIONS

J.D.D., G.L.A.S., C.A.M., and R.M. designed the experiments. J.D.D., G.L.A.S., C.A.M., M.K., and C.L. conducted the experiments. J.D.D., G.L.A.S., C.A.M., M.K., D.B., and R.M. analyzed the data. J.D.D., G.L.A.S., C.A.M., and R.M. wrote the paper. J.D.D., C.A.M., and R.M. acquired funding.

DECLARATION OF INTERESTS

The authors declare no competing interests.

STAR★METHODS

Detailed methods are provided in the online version of this paper and include the following:

- KEY RESOURCES TABLE
- EXPERIMENTAL MODEL AND STUDY PARTICIPANT DETAILS
- METHOD DETAILS
  - BrdU labelling
  - High molecular weight DNA extraction
  - Oxford Nanopore Technology GridION sequencing
  - Processing of Nanopore sequencing files
  - Generation of BrdU scores
  - Generation of ORI densities files
  - Fork speed, fork asymmetry and unidirectional forks
  - Generation of replication forks density files
  - Replication Fork Directionality (RFD) profiles
  - Origin Efficiency Metrics (OEM) profiles
  - SNP density
  - Replication timing
  - Heatmaps, metaplots and graphs
- QUANTIFICATION AND STATISTICAL ANALYSIS

SUPPLEMENTAL INFORMATION

Supplemental information can be found online at https://doi.org/10.1016/j.celrep.2025.116094.

Received: December 3, 2024
Revised: May 21, 2025
Accepted: July 11, 2025
Published: August 5, 2025

REFERENCES

1. Hu, Y., and Stillman, B. (2023). Origins of DNA replication in eukaryotes. Mol. Cell *83*, 352–372. https://doi.org/10.1016/j.molcel.2022.12.024.
2. Costa, A., and Diffley, J.F.X. (2022). The Initiation of Eukaryotic DNA Replication. Annu. Rev. Biochem. *91*, 107–131. https://doi.org/10.1146/annurev-biochem-072321-110228.
3. Nieduszynski, C.A., Knox, Y., and Donaldson, A.D. (2006). Genome-wide identification of replication origins in yeast by comparative genomics. Genes Dev. *20*, 1874–1879. https://doi.org/10.1101/gad.385306.
4. Lee, C.S.K., Weiβ, M., and Hamperl, S. (2023). Where and when to start: Regulating DNA replication origin activity in eukaryotic genomes. Nucleus *14*, 2229642. https://doi.org/10.1080/19491034.2023.2229642.
5. Vouzas, A.E., and Gilbert, D.M. (2023). Replication timing and transcriptional control: beyond cause and effect - part IV. Curr. Opin. Genet. Dev. *79*, 102031. https://doi.org/10.1016/j.gde.2023.102031.
6. Hulke, M.L., Massey, D.J., and Koren, A. (2020). Genomic methods for measuring DNA replication dynamics. Chromosome Res. *28*, 49–67. https://doi.org/10.1007/s10577-019-09624-y.
7. Donaldson, A.D., and Nieduszynski, C.A. (2019). Genome-wide analysis of DNA replication timing in single cells: Yes! We're all individuals. Genome Biol. *20*, 111. https://doi.org/10.1186/s13059-019-1719-y.
8. Muller, C.A., Boemo, M.A., Spingardi, P., Kessler, B.M., Kriaucionis, S., Simpson, J.T., and Nieduszynski, C.A. (2019). Capturing the dynamics of genome replication on individual ultra-long nanopore sequence reads. Nat. Methods *16*, 429–436. https://doi.org/10.1038/s41592-019-0394-y.
9. Hennion, M., Arbona, J.M., Lacroix, L., Cruaud, C., Theulot, B., Tallec, B. L., Proux, F., Wu, X., Novikova, E., Engelen, S., et al. (2020). FORK-seq: replication landscape of the Saccharomyces cerevisiae genome by nanopore sequencing. Genome Biol. *21*, 125. https://doi.org/10.1186/s13059-020-02013-3.

10. Wang, W., Klein, K.N., Proesmans, K., Yang, H., Marchal, C., Zhu, X., Borrman, T., Hastie, A., Weng, Z., Bechhoefer, J., et al. (2021). Genome-wide mapping of human DNA replication by optical replication mapping supports a stochastic model of eukaryotic replication. Mol. Cell *81*, 2975–2988.e6. https://doi.org/10.1016/j.molcel.2021.05.024.

11. Claussin, C., Vazquez, J., and Whitehouse, I. (2022). Single-molecule mapping of replisome progression. Mol. Cell *82*, 1372–1382.e4. https://doi.org/10.1016/j.molcel.2022.02.010.

12. Miura, H., Takahashi, S., Poonperm, R., Tanigawa, A., Takebayashi, S.I., and Hiratani, I. (2019). Single-cell DNA replication profiling identifies spatiotemporal developmental dynamics of chromosome organization. Nat. Genet. *51*, 1356–1368. https://doi.org/10.1038/s41588-019-0474-z.

13. Dileep, V., and Gilbert, D.M. (2018). Single-cell replication profiling to measure stochastic variation in mammalian replication timing. Nat. Commun. *9*, 427. https://doi.org/10.1038/s41467-017-02800-w.

14. Takahashi, S., Miura, H., Shibata, T., Nagao, K., Okumura, K., Ogata, M., Obuse, C., Takebayashi, S.I., and Hiratani, I. (2019). Genome-wide stability of the DNA replication program in single mammalian cells. Nat. Genet. *51*, 529–540. https://doi.org/10.1038/s41588-019-0347-5.

15. Theulot, B., Lacroix, L., Arbona, J.M., Millot, G.A., Jean, E., Cruaud, C., Pellet, J., Proux, F., Hennion, M., Engelen, S., et al. (2022). Genome-wide mapping of individual replication fork velocities using nanopore sequencing. Nat. Commun. *13*, 3295. https://doi.org/10.1038/s41467-022-31012-0.

16. Foss, E.J., Lichauco, C., Gatbonton-Schwager, T., Gonske, S.J., Lofts, B., Lao, U., and Bedalov, A. (2024). Identification of 1600 replication origins in S. cerevisiae. eLife *12*, RP88087. https://doi.org/10.7554/eLife.88087.

17. Carrington, J.T., Wilson, R.H.C., Thiyagarajan, S., Barker, T., Catchpole, L., Durrant, A., Knitlhoffer, V., Watkins, C., Gharbi, K., and Nieduszynski, C.A. (2024). Most human DNA replication initiation is dispersed throughout the genome with only a minority within previously identified initiation zones. Preprint at bioRxiv. https://doi.org/10.1101/2024.04.28.591325.

18. Adl, S.M., Bass, D., Lane, C.E., Lukeš, J., Schoch, C.L., Smirnov, A., Agatha, S., Berney, C., Brown, M.W., Burki, F., et al. (2019). Revisions to the Classification, Nomenclature, and Diversity of Eukaryotes. J. Eukaryot. Microbiol. *66*, 4–119. https://doi.org/10.1111/jeu.12691.

19. Burki, F., Roger, A.J., Brown, M.W., and Simpson, A.G.B. (2020). The New Tree of Eukaryotes. Trends Ecol. Evol. *35*, 43–55. https://doi.org/10.1016/j.tree.2019.08.008.

20. da Silva, M.S., Vitarelli, M.O., Viala, V.L., Tsantarlis, K., da Silva Pires, D., Franco, T.A., de Azevedo, I.L.M.J., Elias, M.C., and Tonelli, R.R. (2023). Clues on the dynamics of DNA replication in Giardia lamblia. J. Cell Sci. *136*, jcs260828. https://doi.org/10.1242/jcs.260828.

21. Zhang, L., Cervantes, M.D., Pan, S., Lindsley, J., Dabney, A., and Kapler, G.M. (2023). Transcriptome analysis of the binucleate ciliate Tetrahymena thermophila with asynchronous nuclear cell cycles. Mol. Biol. Cell *34*, rs1. https://doi.org/10.1091/mbc.E22-08-0326.

22. Matthews, H., Duffy, C.W., and Merrick, C.J. (2018). Checks and balances? DNA replication and the cell cycle in Plasmodium. Parasit. Vectors *11*, 216. https://doi.org/10.1186/s13071-018-2800-1.

23. Castellano, C.M., Lacroix, L., Mathis, E., Prorok, P., Hennion, M., Lopez-Rubio, J.J., Méchali, M., and Gomes, A.R. (2024). The genetic landscape of origins of replication in P. falciparum. Nucleic Acids Res. *52*, 660–676. https://doi.org/10.1093/nar/gkad1103.

24. Totanes, F.I.G., Gockel, J., Chapman, S.E., Bartfai, R., Boemo, M.A., and Merrick, C.J. (2023). A genome-wide map of DNA replication at single-molecule resolution in the malaria parasite Plasmodium falciparum. Nucleic Acids Res. *51*, 2709–2724. https://doi.org/10.1093/nar/gkad093.

25. Butenko, A., Opperdoes, F.R., Flegontova, O., Horák, A., Hampl, V., Keeling, P., Gawryluk, R.M.R., Tikhonenkov, D., Flegontov, P., and Lukeš, J. (2020). Evolution of metabolic capabilities and molecular features of diplonemids, kinetoplastids, and euglenids. BMC Biol. *18*, 23. https://doi.org/10.1186/s12915-020-0754-1.

26. Lukes, J., Butenko, A., Hashimi, H., Maslov, D.A., Votypka, J., and Yurchenko, V. (2018). Trypanosomatids Are Much More than Just Trypanosomes: Clues from the Expanded Family Tree. Trends Parasitol. *34*, 466–480. https://doi.org/10.1016/j.pt.2018.03.002.

27. Devlin, R., Marques, C.A., and McCulloch, R. (2017). Does DNA replication direct locus-specific recombination during host immune evasion by antigenic variation in the African trypanosome? Curr. Genet. *63*, 441–449. https://doi.org/10.1007/s00294-016-0662-7.

28. Marques, C.A., and McCulloch, R. (2018). Conservation and Variation in Strategies for DNA Replication of Kinetoplastid Nuclear Genomes. Curr. Genomics *19*, 98–109. https://doi.org/10.2174/1389202918666170815144627.

29. da Silva, M.S., Pavani, R.S., Damasceno, J.D., Marques, C.A., McCulloch, R., Tosi, L.R.O., and Elias, M.C. (2017). Nuclear DNA Replication in Trypanosomatids: There Are No Easy Methods for Solving Difficult Problems. Trends Parasitol. *33*, 858–874. https://doi.org/10.1016/j.pt.2017.08.002.

30. Tiengwe, C., Marques, C.A., and McCulloch, R. (2014). Nuclear DNA replication initiation in kinetoplastid parasites: new insights into an ancient process. Trends Parasitol. *30*, 27–36. https://doi.org/10.1016/j.pt.2013.10.009.

31. Rocha-Granados, M.C., and Klingbeil, M.M. (2016). Leishmania DNA Replication Timing: A Stochastic Event? Trends Parasitol. *32*, 755–757. https://doi.org/10.1016/j.pt.2016.05.011.

32. Damasceno, J.D., Marques, C.A., Black, J., Briggs, E., and McCulloch, R. (2021). Read, Write, Adapt: Challenges and Opportunities during Kinetoplastid Genome Replication. Trends Genet. *37*, 21–34. https://doi.org/10.1016/j.tig.2020.09.002.

33. Tiengwe, C., Marcello, L., Farr, H., Dickens, N., Kelly, S., Swiderski, M., Vaughan, D., Gull, K., Barry, J.D., Bell, S.D., and McCulloch, R. (2012). Genome-wide analysis reveals extensive functional interaction between DNA replication initiation and transcription in the genome of Trypanosoma brucei. Cell Rep. *2*, 185–197. https://doi.org/10.1016/j.celrep.2012.06.007.

34. Devlin, R., Marques, C.A., Paape, D., Prorocic, M., Zurita-Leal, A.C., Campbell, S.J., Lapsley, C., Dickens, N., and McCulloch, R. (2016). Mapping replication dynamics in Trypanosoma brucei reveals a link with telomere transcription and antigenic variation. eLife *5*, e12765. https://doi.org/10.7554/eLife.12765.

35. Krasiļņikova, M., Marques, C.A., Briggs, E.M., Lapsley, C., Hamilton, G., Beraldi, D., Crouch, K., and McCulloch, R. (2024). Nanopore sequencing reveals that DNA replication compartmentalisation dictates genome stability and instability in Trypanosoma brucei. Preprint at bioRxiv. https://doi.org/10.1101/2024.05.07.592375.

36. de Araujo, C.B., da Cunha, J.P.C., Inada, D.T., Damasceno, J., Lima, A.R.J., Hiraiwa, P., Marques, C., Gonçalves, E., Nishiyama-Junior, M.Y., McCulloch, R., and Elias, M.C. (2020). Replication origin location might contribute to genetic variability in Trypanosoma cruzi. BMC Genom. *21*, 414. https://doi.org/10.1186/s12864-020-06803-8.

37. Vitarelli, M.d.O., Franco, T.A., Pires, D.d.S., Lima, A.R.J., Viala, V.L., Kraus, A.J., de Azevedo, I.d.L.M.J., da Cunha, J.P.C., and Elias, M.C. (2024). Integrating high-throughput analysis to create an atlas of replication origins in *Trypanosoma cruzi* in the context of genome structure and variability. mBio *15*, e0031924–00324. https://doi.org/10.1128/mbio.00319-24.

38. Marques, C.A., Dickens, N.J., Paape, D., Campbell, S.J., and McCulloch, R. (2015). Genome-wide mapping reveals single-origin chromosome replication in Leishmania, a eukaryotic microbe. Genome Biol. *16*, 230. https://doi.org/10.1186/s13059-015-0788-9.

39. Damasceno, J.D., Marques, C.A., Beraldi, D., Crouch, K., Lapsley, C., Obonaga, R., Tosi, L.R., and McCulloch, R. (2020). Genome duplication in Leishmania major relies on persistent subtelomeric DNA replication. eLife *9*, e58030. https://doi.org/10.7554/eLife.58030.

40. Damasceno, J.D., Reis-Cunha, J., Crouch, K., Beraldi, D., Lapsley, C., Tosi, L.R.O., Bartholomeu, D., and McCulloch, R. (2020). Conditional knockout of RAD51-related genes in Leishmania major reveals a critical

role for homologous recombination during genome replication. PLoS Genet. *16*, e1008828. https://doi.org/10.1371/journal.pgen.1008828.

41. Clayton, C. (2019). Regulation of gene expression in trypanosomatids: living with polycistronic transcription. Open Biol. *9*, 190072. https://doi.org/10.1098/rsob.190072.
42. El-Sayed, N.M., Myler, P.J., Blandin, G., Berriman, M., Crabtree, J., Aggarwal, G., Caler, E., Renauld, H., Worthey, E.A., Hertz-Fowler, C., et al. (2005). Comparative genomics of trypanosomatid parasitic protozoa. Science *309*, 404–409. https://doi.org/10.1126/science.1112181.
43. Reis-Cunha, J.L., Pimenta-Carvalho, S.A., Almeida, L.V., Coqueiro-Dos-Santos, A., Marques, C.A., Black, J.A., Damasceno, J., McCulloch, R., Bartholomeu, D.C., and Jeffares, D.C. (2024). Ancestral aneuploidy and stable chromosomal duplication resulting in differential genome structure and gene expression control in trypanosomatid parasites. Genome Res. *34*, 441–453. https://doi.org/10.1101/gr.278550.123.
44. Callejas, S., Leech, V., Reitter, C., and Melville, S. (2006). Hemizygous subtelomeres of an African trypanosome chromosome may account for over 75% of chromosome length. Genome Res. *16*, 1109–1118.
45. Berriman, M., Ghedin, E., Hertz-Fowler, C., Blandin, G., Renauld, H., Bartholomeu, D.C., Lennard, N.J., Caler, E., Hamlin, N.E., Haas, B., et al. (2005). The genome of the African trypanosome Trypanosoma brucei. Science *309*, 416–422.
46. Cosentino, R.O., Brink, B.G., and Siegel, T.N. (2021). Allele-specific assembly of a eukaryotic genome corrects apparent frameshifts and reveals a lack of nonsense-mediated mRNA decay. NAR Genom. Bioinform. *3*, lqab082. https://doi.org/10.1093/nargab/lqab082.
47. Muller, L.S.M., Cosentino, R.O., Forstner, K.U., Guizetti, J., Wedel, C., Kaplan, N., Janzen, C.J., Arampatzi, P., Vogel, J., Steinbiss, S., et al. (2018). Genome organization and DNA accessibility control antigenic variation in trypanosomes. Nature *563*, 121–125. https://doi.org/10.1038/s41586-018-0619-8.
48. Batrakou, D.G., Müller, C.A., Wilson, R.H.C., and Nieduszynski, C.A. (2020). DNA copy-number measurement of genome replication dynamics by high-throughput sequencing: the sort-seq, sync-seq and MFA-seq family. Nat. Protoc. *15*, 1255–1284. https://doi.org/10.1038/s41596-019-0287-7.
49. Kim, H.S. (2019). Genome-wide function of MCM-BP in Trypanosoma brucei DNA replication and transcription. Nucleic Acids Res. *47*, 634–647. https://doi.org/10.1093/nar/gky1088.
50. Kim, H.S. (2021). Genetic Interaction Between Site-Specific Epigenetic Marks and Roles of H4v in Transcription Termination in Trypanosoma brucei. Front. Cell Dev. Biol. *9*, 744878. https://doi.org/10.3389/fcell.2021.744878.
51. Marques, C.A., Tiengwe, C., Lemgruber, L., Damasceno, J.D., Scott, A., Paape, D., Marcello, L., and McCulloch, R. (2016). Diverged composition and regulation of the Trypanosoma brucei origin recognition complex that mediates DNA replication initiation. Nucleic Acids Res. *44*, 4763–4784. https://doi.org/10.1093/nar/gkw147.
52. Tiengwe, C., Marcello, L., Farr, H., Gadelha, C., Burchmore, R., Barry, J. D., Bell, S.D., and McCulloch, R. (2012). Identification of ORC1/CDC6-Interacting Factors in Trypanosoma brucei Reveals Critical Features of Origin Recognition Complex Architecture. PLoS One *7*, e32674.
53. Godoy, P.D.d.M., Nogueira-Junior, L.A., Paes, L.S., Cornejo, A., Martins, R.M., Silber, A.M., Schenkman, S., and Elias, M.C. (2009). Trypanosome prereplication machinery contains a single functional orc1/cdc6 protein, which is typical of archaea. Eukaryot. Cell *8*, 1592–1603.
54. Garcia-Silva, M.R., Sollelis, L., MacPherson, C.R., Stanojcic, S., Kuk, N., Crobu, L., Bringaud, F., Bastien, P., Pagès, M., Scherf, A., and Sterkers, Y. (2017). Identification of the centromeres of Leishmania major: revealing the hidden pieces. EMBO Rep. *18*, 1968–1977. https://doi.org/10.15252/embr.201744216.
55. Stanojcic, S., Sollelis, L., Kuk, N., Crobu, L., Balard, Y., Schwob, E., Bastien, P., Pagès, M., and Sterkers, Y. (2016). Single-molecule analysis of DNA replication reveals novel features in the divergent eukaryotes Leishmania and Trypanosoma brucei versus mammalian cells. Sci. Rep. *6*, 23142. https://doi.org/10.1038/srep23142.
56. Lombrana, R., Alvarez, A., Fernandez-Justel, J.M., Almeida, R., Poza-Carrion, C., Gomes, F., Calzada, A., Requena, J.M., and Gomez, M. (2016). Transcriptionally Driven DNA Replication Program of the Human Parasite Leishmania major. Cell Rep. *16*, 1774–1786. https://doi.org/10.1016/j.celrep.2016.07.007.
57. Lombrana, R., Alvarez, A., Fernandez-Justel, J.M., Almeida, R., Poza-Carrion, C., Gomes, F., Calzada, A., Requena, J.M., and Gomez, M. (2016). Transcriptionally Driven DNA Replication Program of the Human Parasite Leishmania major. Cell Rep. *16*, 1774–1786. https://doi.org/10.1016/j.celrep.2016.07.007.
58. Ubeda, J.M., Raymond, F., Mukherjee, A., Plourde, M., Gingras, H., Roy, G., Lapointe, A., Leprohon, P., Papadopoulou, B., Corbeil, J., and Ouellette, M. (2014). Genome-wide stochastic adaptive DNA amplification at direct and inverted DNA repeats in the parasite Leishmania. PLoS Biol. *12*, e1001868. https://doi.org/10.1371/journal.pbio.1001868.
59. Foulk, M.S., Urban, J.M., Casella, C., and Gerbi, S.A. (2015). Characterizing and controlling intrinsic biases of lambda exonuclease in nascent strand sequencing reveals phasing between nucleosomes and G-quadruplex motifs around a subset of human replication origins. Genome Res. *25*, 725–735. https://doi.org/10.1101/gr.183848.114.
60. Marsico, G., Chambers, V.S., Sahakyan, A.B., McCauley, P., Boutell, J.M., Antonio, M.D., and Balasubramanian, S. (2019). Whole genome experimental maps of DNA G-quadruplexes in multiple species. Nucleic Acids Res. *47*, 3862–3874. https://doi.org/10.1093/nar/gkz179.
61. Boemo, M.A. (2021). DNAscent v2: detecting replication forks in nanopore sequencing data with deep learning. BMC Genom. *22*, 430. https://doi.org/10.1186/s12864-021-07736-6.
62. Assis, L.H.d.C., de Paiva, S.C., and Cano, M.I.N. (2023). Behind Base J: The Roles of JBP1 and JBP2 on Trypanosomatids. Pathogens *12*, 467. https://doi.org/10.3390/pathogens12030467.
63. Theulot, B., Tourancheau, A., Simonin Chavignier, E., Jean, E., Arbona, J. M., Audit, B., Hyrien, O., Lacroix, L., and Le Tallec, B. (2025). Telomere-to-telomere DNA replication timing profiling using single-molecule sequencing with Nanotiming. Nat. Commun. *16*, 242. https://doi.org/10.1038/s41467-024-55520-3.
64. Damasceno, J.D., Briggs, E.M., Krasilnikova, M., Marques, C.A., Lapsley, C., and McCulloch, R. (2024). R-loops acted on by RNase H1 are a determinant of chromosome length-associated DNA replication timing and genome stability in Leishmania. Preprint at bioRxiv. https://doi.org/10.1101/2024.04.29.591643.
65. Hennion, M., Theulot, B., Arbona, J.M., Audit, B., and Hyrien, O. (2022). FORK-seq: Single-Molecule Profiling of DNA Replication. Methods Mol. Biol. *2477*, 107–128. https://doi.org/10.1007/978-1-0716-2257-5_8.
66. Liu, Y., Wu, X., d'Aubenton-Carafa, Y., Thermes, C., and Chen, C.L. (2023). OKseqHMM: a genome-wide replication fork directionality analysis toolkit. Nucleic Acids Res. *51*, e22. https://doi.org/10.1093/nar/gkac1239.
67. Wu, X., Liu, Y., d'Aubenton-Carafa, Y., Thermes, C., Hyrien, O., Chen, C. L., and Petryk, N. (2023). Genome-wide measurement of DNA replication fork directionality and quantification of DNA replication initiation and termination with Okazaki fragment sequencing. Nat. Protoc. *18*, 1260–1295. https://doi.org/10.1038/s41596-022-00793-5.
68. McGuffee, S.R., Smith, D.J., and Whitehouse, I. (2013). Quantitative, genome-wide analysis of eukaryotic replication initiation and termination. Mol. Cell *50*, 123–135. https://doi.org/10.1016/j.molcel.2013.03.004.
69. Petryk, N., Kahli, M., d'Aubenton-Carafa, Y., Jaszczyszyn, Y., Shen, Y., Silvain, M., Thermes, C., Chen, C.L., and Hyrien, O. (2016). Replication landscape of the human genome. Nat. Commun. *7*, 10208. https://doi.org/10.1038/ncomms10208.
70. Tsankov, A., Yanagisawa, Y., Rhind, N., Regev, A., and Rando, O.J. (2011). Evolutionary divergence of intrinsic and trans-regulated nucleosome

positioning sequences reveals plastic rules for chromatin organization. Genome Res. *21*, 1851–1862. https://doi.org/10.1101/gr.122267.111.

71. Grunebast, J., Lorenzen, S., and Clos, J. (2025). Genome-wide quantification of polycistronic transcription in Leishmania major. mBio *16*, e0224124. https://doi.org/10.1128/mbio.02241-24.
72. Damasceno, J.D., Briggs, E.M., Krasilnikova, M., Marques, C.A., Lapsley, C., and McCulloch, R. (2025). R-loops acted on by RNase H1 influence DNA replication timing and genome stability in Leishmania. Nat. Commun. *16*, 1470. https://doi.org/10.1038/s41467-025-56785-y.
73. Briggs, E., Hamilton, G., Crouch, K., Lapsley, C., and McCulloch, R. (2018). Genome-wide mapping reveals conserved and diverged R-loop activities in the unusual genetic landscape of the African trypanosome genome. Nucleic Acids Res. *46*, 11789–11805. https://doi.org/10.1093/nar/gky928.
74. Murat, P., Perez, C., Crisp, A., van Eijk, P., Reed, S.H., Guilbaud, G., and Sale, J.E. (2022). DNA replication initiation shapes the mutational landscape and expression of the human genome. Sci. Adv. *8*, eadd3686. https://doi.org/10.1126/sciadv.add3686.
75. Hoggard, T., Shor, E., Müller, C.A., Nieduszynski, C.A., and Fox, C.A. (2013). A Link between ORC-origin binding mechanisms and origin activation time revealed in budding yeast. PLoS Genet. *9*, e1003798. https://doi.org/10.1371/journal.pgen.1003798.
76. Muller, C.A., Hawkins, M., Retkute, R., Malla, S., Wilson, R., Blythe, M.J., Nakato, R., Komata, M., Shirahige, K., de Moura, A.P., and Nieduszynski, C.A. (2014). The dynamics of genome replication using deep sequencing. Nucleic Acids Res. *42*, e3. https://doi.org/10.1093/nar/gkt878.
77. Muller, C.A., and Nieduszynski, C.A. (2012). Conservation of replication timing reveals global and local regulation of replication origin activity. Genome Res. *22*, 1953–1962. https://doi.org/10.1101/gr.139477.112.
78. Geoghegan, V., Carnielli, J.B.T., Jones, N.G., Saldivia, M., Antoniou, S., Hughes, C., Neish, R., Dowle, A., and Mottram, J.C. (2022). CLK1/CLK2-driven signalling at the Leishmania kinetochore is captured by spatially referenced proximity phosphoproteomics. Commun. Biol. *5*, 1305. https://doi.org/10.1038/s42003-022-04280-1.
79. da Silva, M.S., Cayres-Silva, G.R., Vitarelli, M.O., Marin, P.A., Hiraiwa, P. M., Araújo, C.B., Scholl, B.B., Ávila, A.R., McCulloch, R., Reis, M.S., and Elias, M.C. (2019). Transcription activity contributes to the firing of non-constitutive origins in African trypanosomes helping to maintain robustness in S-phase duration. Sci. Rep. *9*, 18512. https://doi.org/10.1038/s41598-019-54366-w.
80. Gros, J., Devbhandari, S., and Remus, D. (2014). Origin plasticity during budding yeast DNA replication in vitro. The EMBO journal *33*, 621–636. https://doi.org/10.1002/embj.201387278.
81. Kurth, I., and Gautier, J. (2010). Origin-dependent initiation of DNA replication within telomeric sequences. Nucleic Acids Res. *38*, 467–476.
82. On, K.F., Beuron, F., Frith, D., Snijders, A.P., Morris, E.P., and Diffley, J.F. X. (2014). Prereplicative complexes assembled in vitro support origin-dependent and independent DNA replication. The EMBO journal *33*, 605–620. https://doi.org/10.1002/embj.201387369.
83. Hawkins, M., Malla, S., Blythe, M.J., Nieduszynski, C.A., and Allers, T. (2013). Accelerated growth in the absence of DNA replication origins. Nature *503*, 544–547. https://doi.org/10.1038/nature12650.
84. Shibata, E., Kiran, M., Shibata, Y., Singh, S., Kiran, S., and Dutta, A. (2016). Two subunits of human ORC are dispensable for DNA replication and proliferation. eLife *5*, e19084. https://doi.org/10.7554/eLife.19084.
85. Li, H. (2018). Minimap2: pairwise alignment for nucleotide sequences. Bioinformatics *34*, 3094–3100. https://doi.org/10.1093/bioinformatics/bty191.
86. Koster, J., and Rahmann, S. (2012). Snakemake–a scalable bioinformatics workflow engine. Bioinformatics *28*, 2520–2522. https://doi.org/10.1093/bioinformatics/bts480.
87. Danecek, P., Bonfield, J.K., Liddle, J., Marshall, J., Ohan, V., Pollard, M.O., Whitwham, A., Keane, T., McCarthy, S.A., Davies, R.M., and Li, H. (2021). Twelve years of SAMtools and BCFtools. GigaScience *10*, giab008. https://doi.org/10.1093/gigascience/giab008.
88. Galaxy Community (2024). The Galaxy platform for accessible, reproducible, and collaborative data analyses: 2024 update. Nucleic Acids Res. *52*, W83–W94. https://doi.org/10.1093/nar/gkae410.

STAR★METHODS

KEY RESOURCES TABLE

REAGENT or RESOURCE	SOURCE	IDENTIFIER
Chemicals, peptides, and recombinant proteins		
HOMEM medium (Dulbecco's Modified Eagle Medium (DMEM))	Merck	D6429
Fetal Bovine Serum (FBS), Heat Inactivated	Gibco	A5670801
5-Bromo-2′-deoxyuridine (BrdU)	Sigma	B5002
Thymidine	Merck	T1895
Hydroxyurea	Sigma	H8627
Critical commercial assays		
MagAttract High Molecular Weight DNA Kit	QIAGEN	67563
Ligation Sequencing Kit	Oxford Nanopore Technologies	SQK-LSK110
R9.4.1 GridION flow cells	Oxford Nanopore Technologies	FLO-MIN106D
Deposited data		
Nanopore Sequencing Data	This study	PRJEB82099
Experimental models: Organisms/strains		
Leishmania major v9		MHOM/IL/80/Friedlin
Software and algorithms		
Guppy basecaller v6.4.2	Oxford Nanopore Technologies	https://nanoporetech.com/software/other/guppy
TriTrypDB	TriTrypDB	https://tritrypdb.org/tritrypdb/app
Mnimap2	Li et al., 2018[84]	https://github.com/lh3/minimap2
Samtools	Danecek et al., 2021[85]	https://github.com/samtools/samtools
DNAscent v2	Boemo, 2021[61]	https://github.com/MBoemo/DNAscent
Galaxy	Galaxy Team[86]	https://usegalaxy.org
R	R Core Team	http://www.r-project.org/
GraphPad Prism 10.2.2	Graphpad	https://www.graphpad.com

EXPERIMENTAL MODEL AND STUDY PARTICIPANT DETAILS

Promastigotes derived from *Leishmania major* V9 (MHOM/IL/80/Friedlin) strain were cultured at 26°C in HOMEM medium supplemented with 10% heat-inactivated foetal bovine serum.

METHOD DETAILS

BrdU labelling

Prior to labelling, parasites were seeded at 5×10^5 cells.mL^{-1} and allowed to proliferate until exponentially growing phase at $\sim 5 \times 10^6$ cells.mL^{-1}. Parasites were exposed to BrdU for a total of 5 min. First, cells were incubated with 150 μM BrdU at 26°C for 2 min and centrifuged at 2,300 g for 2 min. Removal of tubes from centrifuge and discarding of BrdU-containing medium took approximately 1 min. Cell pellets were resuspended in culturing medium supplemented with 1 mM thymidine and incubated at 26°C for 1 h. Then, cells were collected by centrifugation at 2,300 g for 2 min and pellets were stored at −20°C until used. The same labelling approach was used for synchronised cells after treatment with hydroxyurea (as described in the main text).

High molecular weight DNA extraction

To ensure isolation of long DNA molecules, genomic DNA extractions were performed using the MagAttract High Molecular Weight DNA Kit (QIAGEN). For each extraction, a pellet containing approximately 5×10^8 cells was removed from −20°C storage and immediately resuspended in lysis buffer. All further processing steps were performed following the manufacturers' instructions. After elution, DNA samples were incubated at 4°C for 24 to 48 h to allow complete sample homogenisation.

Oxford Nanopore Technology GridION sequencing

High molecular weight genomic DNA samples were subjected to library preparation for Nanopore sequencing using the Ligation Sequencing Kit SQK-LSK110 (Oxford Nanopore Technologies). Approximately 5 μg input genomic DNA was used in each reaction and all processing steps were performed following the manufacturers' instructions. Libraries were loaded onto R9.4.1 GridION flow cells (Oxford Nanopore Technologies) and sequenced for up to 48 h. When needed, sequencing was paused, flow cells washed and reloaded with extra library material.

Processing of Nanopore sequencing files

The Nanopore run directories were processed with *Guppy basecaller v6.4.2* with configuration *dna_r9.4.1_450bps_fast.cfg*. The resulting fastq files were aligned to the reference genome *Leishmania major Friedlin* v45 (https://tritrypdb.org/tritrypdb/app) using *minimap2*[85] with setting *-x map-ont*. The resulting bam files were sorted and indexed with *samtools*.[87] Sorted bam files were subjected to BrdU calling with the *DNAscent detect* function from *DNAscent v2*[61] (https://github.com/MBoemo/DNAscent - commit 7e4be09) using commands *index, detect* and *forkSense*. Output files from *DNAscent detect* contained the probability of BrdU at each thymidine in the genome. These files were rearranged to a bedgraph-like format containing six columns: chrom, start, end, percentage of reads with BrdU calls, read depth and number of reads with BrdU calls. The output from *DNAscent forkSense* contained coordinates of left- and right-moving forks as well as for the ORI and TER sites. These files were also rearranged to a bedgraph-like format containing nine columns: chrom, start, end, probability of left-moving fork, probability of right-moving fork, read name, strand, alignment start and alignment end. This workflow was run within a mamba environment and implemented as a Snakemake[86] pipeline as detailed here: https://github.com/glaParaBio/dnascent-fork-detection.

Generation of BrdU scores

Output files from *DNAscent detect* were processed to remove all BrdU calls with probability <0.5 whilst retaining BrdU calls with probability ≥0.5. Retained BrdU calls were averaged into 500 bp sequential windows across the genome using *bedtools MapBed* on *Galaxy* (https://usegalaxy.org/).[88] The scores from the resulting bedgraph files were converted into z-scores calculated in rolling windows of 15 kb by using R.

Generation of ORI densities files

Given the BrdU pulse length (2 min) and the average fork speed in *L. major* (2.5 kb.min^{-1}), we expect ORIs fired immediately before labelling to have maximum length of 10 Kb. Therefore, output files from *DNAscent forkSense* were processed to retain only ORI calls ≤10 kb. Midpoints for retained ORIs were determined and *bedtools MapBed* was used to count the number of midpoints in 5000 bp sequential windows across the genome and the resulting bedgraph files were converted into biwig. Then, *bigwigCompare* was used to calculate the ration between the number of ORIs and the coverage in each 5000 bp window. The resulting files contains the *ORI Usage* files and were used to compare global changes and patterns between conditions (Figures 2D and 2E). *ORI Usage* files were further processed by calculating z-scores in rolling windows of 50 kb using R. These are the Relative *ORI Usage* and were used to compare local changes and patterns between conditions (Figures 2B, 2F and 2G).

Fork speed, fork asymmetry and unidirectional forks

Only forks with lengths equal to or greater than 5 kb and positioned at least 5 kb away from alignment boundaries were included in these analyses. Fork speed was determined by dividing the fork length (in kb) by the duration of BrdU exposure (5 min). Fork asymmetry was defined as the ratio between the lengths (in kb) of the longer and shorter forks flanking an ORI call. Forks were classified as unidirectional if they were located at a distance greater than 10 kb from any ORI or TER site and at least 10 kb apart from any other fork event.

Generation of replication forks density files

Output files from *DNAscent forkSense* were processed to remove fork calls with probability <0.5 whilst retaining fork calls with probability ≥0.5 in reads with length of at least 15 kb. Density of retained forks calls calculated into 20 bp sequential windows across the genome using *bedtools MapBed*, further normalised against the sequencing coverage using *bigwigCompare* and scaled to 0 and 1 using R. In this way, two bedgraph files, one with the right-moving forks and another with the left-moving forks, were generated.

Replication Fork Directionality (RFD) profiles

Bedgraph files with the normalised density of right- and left-moving forks were used as input for determining RFD in 1kb rolling windows with 50bp steps. RFD has been previously defined[66,67,69] and was calculated as ((right-moving forks – left-moving-forks)/(right-moving forks + left-moving-forks)).

Origin Efficiency Metrics (OEM) profiles

Bedgraph files with the normalised density of right- and left-moving forks were used as input. OEM was calculated in 10 kb rolling windows with 50bp steps. The rolling window was divided into 5 kb left-window and 5 kb right-window as previously defined.[66–68] Calculations were performed as following: $[(\text{right-moving forks})/(\text{right-moving forks} + \text{left-moving-forks})]^{\textit{Left-window}}$ - $[(\text{right-moving forks})/(\text{right-moving forks} + \text{left-moving-forks})]^{\textit{Right-window}}$.

SNP density

Sequencing files of three independently cultivated *Leishmania major* strain LT252 (MHOM/IR/1983/IR) were obtained from.[40] SNPs were called using *FreeBayes* on *Galaxy*. Only SNPs with at least two supporting reads and with QUAL >20 were retained. SNPs counts were averaged into 1 kb sequential windows across the genome using *bedtools MapBed* and then normalised against sequencing coverage by using *bigwigCompare*.

Replication timing

Marker Frequency Analysis after whole genome sequencing (MFA-seq) was used to generate replication timing profiles, as previously described.[39] Briefly, read depth from an asynchronous exponentially growing culture was compared with read depth from a non-dividing, stationary phase culture.

Heatmaps, metaplots and graphs

Heatmaps and metaplots were generated with *deepTools plotHeatmap* and *plotProfile* tools, respectively, on Galaxy. Remaining graphs and associated statistical analysis were generated using Prism GraphPad.

QUANTIFICATION AND STATISTICAL ANALYSIS

Each experiment was repeated independently two times. All quantification and statistical analysis were performed using GraphPad Prism v10.0. Statistical test used are indicated in the legends of each Figure. *p* values and other relevant values are shown in each figure. Box plots: horizontal line indicates median, cross indicate mean. Metaplots: line indicates mean, shaded area indicate ±SEM. Simple linear regression: line indicates best fit, shaded areas represent 95% confidence intervals, circles represent mean values.

Cell Reports, Volume *44*

Supplemental information

Leishmania major chromosomes are replicated from a single high-efficiency locus supplemented by thousands of lower efficiency initiation events

Jeziel D. Damasceno, Gabriel L.A. Silva, Catarina A. Marques, Marija Krasilnikova, Craig Lapsley, Dario Beraldi, and Richard McCulloch

Figure S1

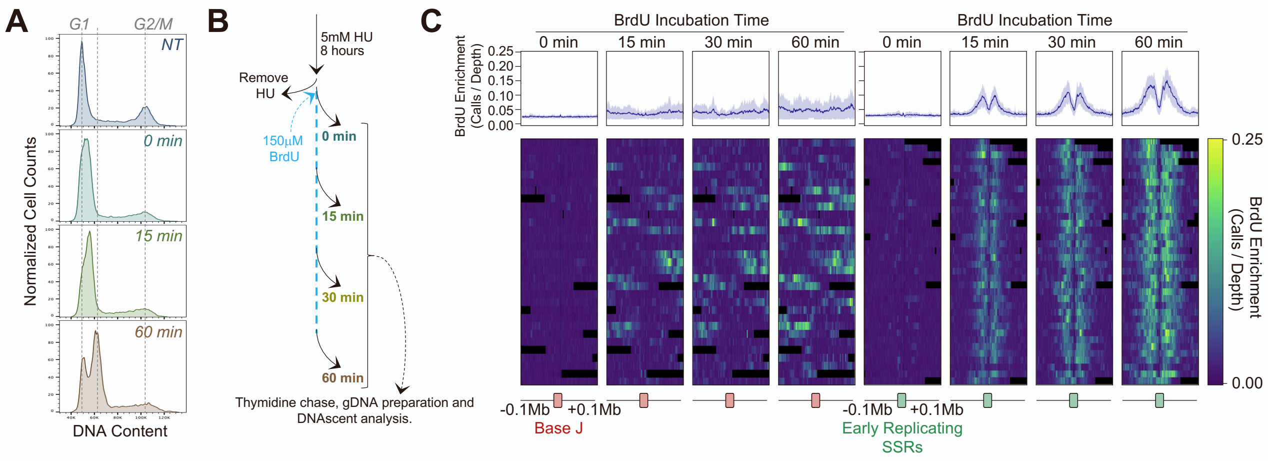

Figure S1. Testing incorporation of BrdU into *Leishmania major* promastigote nuclear DNA, related to Figure 1. A) Exponentially growing *L. major* promastigotes were left untreated (NT) or incubated with HU for 8 hrs to arrest cell cycle, transferred into fresh HU-free medium and collected at the indicated later time points. FACS analysis was used to assess DNA content profile. **B)** Schematic of the experimental approach used for analysis show in C. Exponentially growing *L. major* promastigotes were treated with HU for 8 hrs, then transferred into fresh HU-free medium containing 150 µM BrdU and collected 0, 15, 30 and 60 min later. For each sample, 1 mM thymidine was added for 1 hour, then high molecular weight DNA was extracted and subjected to Oxford Nanopore Technologies sequencing. **C)** Summary plots (top) and colormaps (bottom) comparing BrdU calls density (as normalised by the sequencing depth) between regions of base J accumulation and early replicating SSRs.

Figure S2

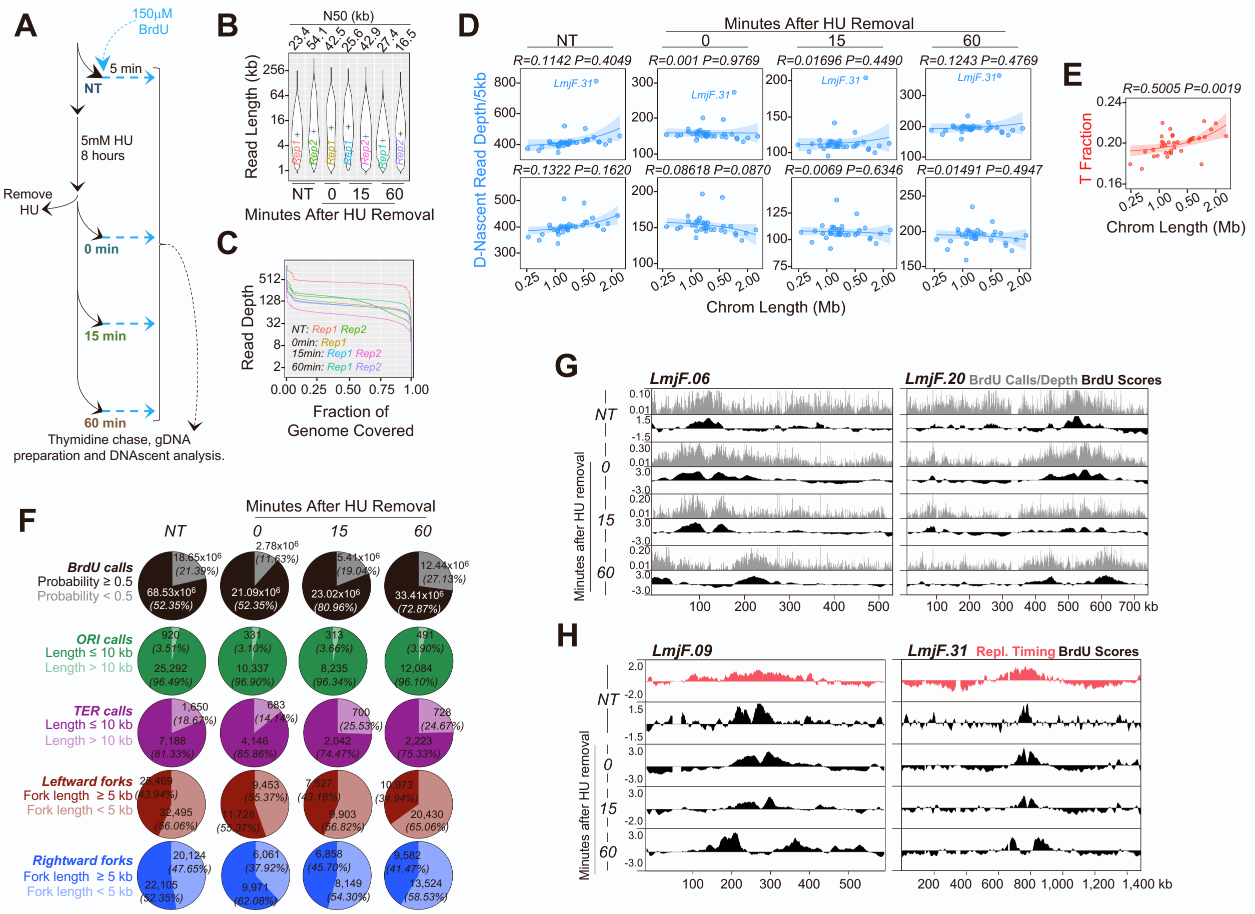

Figure S2. Comparing sequencing yield and BrdU incorporation profiles between NT and HU treated samples, related to Figure 1. A) Schematic of the experimental approach used for all the remaining analysis presented in main and supplementary figures. Exponentially growing *L. major* promastigotes were treated with HU for 8 hrs, then moved into fresh HU-free medium. Cells were collected 0, 15 and 60 min later, then incubated with 150 μM BrdU for 5 minutes followed by 1 mM thymidine chase for 1 hour. High molecular weight DNA was extracted and subjected to Oxford Nanopore Technologies sequencing and analysis with DNAscent. **B)** and **C)** Read length distribution and genome coverage, respectively, from the indicated replicates (Rep) and condition. **D)** and **E)** Simple linear regression analysis comparing average read depth or T content, respectively, from each chromosome with its length. In G, chromosomes 31, which is estimated to be tetraploid, was either included (top) or excluded (bottom) from the analysis. Shaded areas represent 95% confidence intervals. *R* and *P* values are indicated at the top of each panel. **F)** Proportion of retained (darker coloured) and discarded (light coloured) of the indicated features after filtering based on read length (>10kb) and mapping quality (>20). **G)** Snapshots showing distribution BrdU along the entire indicated chromosomes in NT cells and after HU synchronisation. In grey, BrdU signal expressed as the number of calls normalised by the sequencing depth. In black, BrdU score which is the z-scores of calls normalised by the read depth. **H)** Snapshots showing BrdU scores (black) in the entire indicated chromosomes in NT cells and after HU synchronisation. Top track (salmon), DNA replication timing profile as determined by MFA-seq in NT cells.

Figure S3

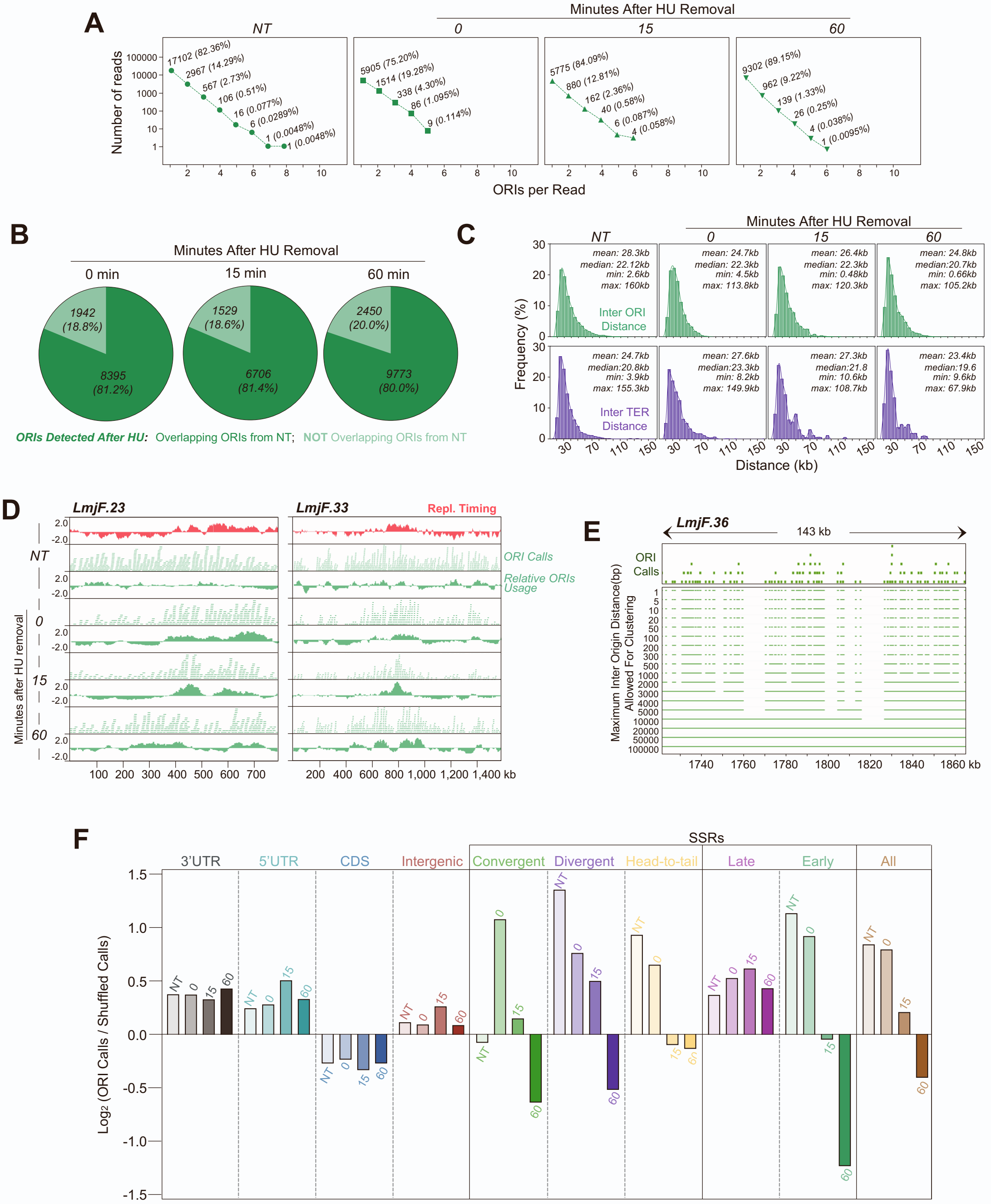

Figure S3. Genome-wide distribution of ORIs detected by DNAscent, related to Figure 2. A) Number and proportion of reads with the indicated number of ORIs from NT and HU synchronised cells. **B)** Genome-wide quantification of the overlap between ORIs from HU treated cells and NT cells. **C)** Frequency distribution of distances between pairs of ORIs or TERs detected in the same single DNA molecule from NT and HU synchronised cells. **D)** Snapshots showing ORIs distribution (green) in the entire indicated chromosomes in NT and HU synchronised cells. ORI calls as detected at the single molecule level is shown as the first green track of each condition. Relative ORIs usage expressed as the z-scores of the number of calls in 5 kb windows normalised by the sequencing depth is shown as the second green track of each condition. Top track (salmon), DNA replication timing profile as determined by MFA-seq in NT cells. **E)** Clustering of DNAscent ORI calls. A window of 143 kb from chromosome 36 showing clusters generated when allowing the indicated maximum distance between ORI calls. Each horizontal green bar indicate an individual cluster. The extension of each green bar is referred to as cluster width. **F)** Distribution of DNAscent ORI calls in the indicated annotated genomic features from *L. major* in NT cells and at the indicated time points after HU removal.

Figure S4

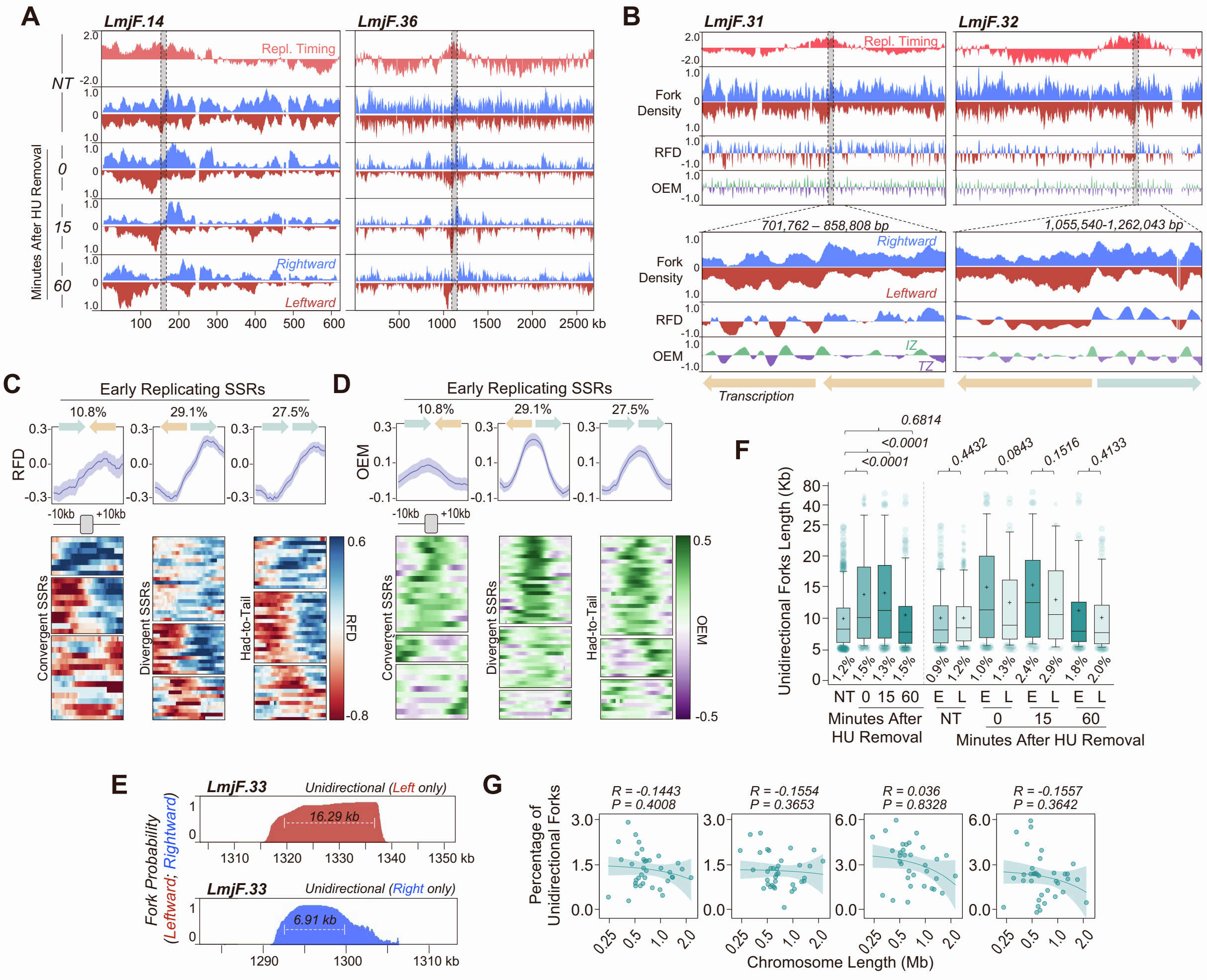

Figure S4. Analysis of DNA replication forks detected by DNAscent, related to Figure 4. A) Snapshots showing density of normalised leftwards (red) and rightwards (blue) moving forks in the entire indicated chromosomes in NT and HU treated cells. Top track (salmon), DNA replication timing profile as determined by MFA-seq in NT cells. Grey vertical bar, position of the single early replicating SSR. **B)** Snapshots showing normalised density of leftwards (red) and rightwards (blue) moving forks and the corresponding RFD and OEM profiles for the entire indicated chromosomes in NT cells. Magnification around the single early replicating SSR is displayed. Arrows at the bottom indicate transcription direction in the PTUs flanking the early replicating SSRs. **C)** and **D)** Summary plots (top) and colourmaps (bottom) comparing RFD and OEM profiles, respectively, around the indicated group of SSRs in NT cells. SSRs were grouped according to the transcription direction from their flanking PTUs: convergent SSRs, where transcription from upstream and downstream PTUs terminates; divergent SSRs, where transcription of upstream and downstream PTUs initiates; head-to-tail SSRs, where transcription from the upstream and downstream PTUs terminates and initiates, respectively. The fraction of SSRs that are predicted by MFA-seq to be early replicating in each group is shown at the top. **E)** Representative Nanopore individual reads showing reads in which only leftwards and rightwards moving forks were detected. Leftwards and rightwards moving replication forks probabilities are indicated as red and blue, respectively. White dotted horizontal lines indicate the position of replication forks calls. **F)** Comparing the length of unidirectional forks between NT and HU treated cells. The percentages of unidirectional forks relative to the total number of left and right moving forks is indicated bellow each box plot. E and L, early and late replicating compartments. *P* values are indicated at the top. Statistical test, Kruskal-Wallis. **G)** Simple linear regression between chromosome length and the average percentage of unidirectional forks in each chromosome. Shaded areas represent 95% confidence intervals. *R* and *P* values are indicated at the top of each panel.

Figure S5

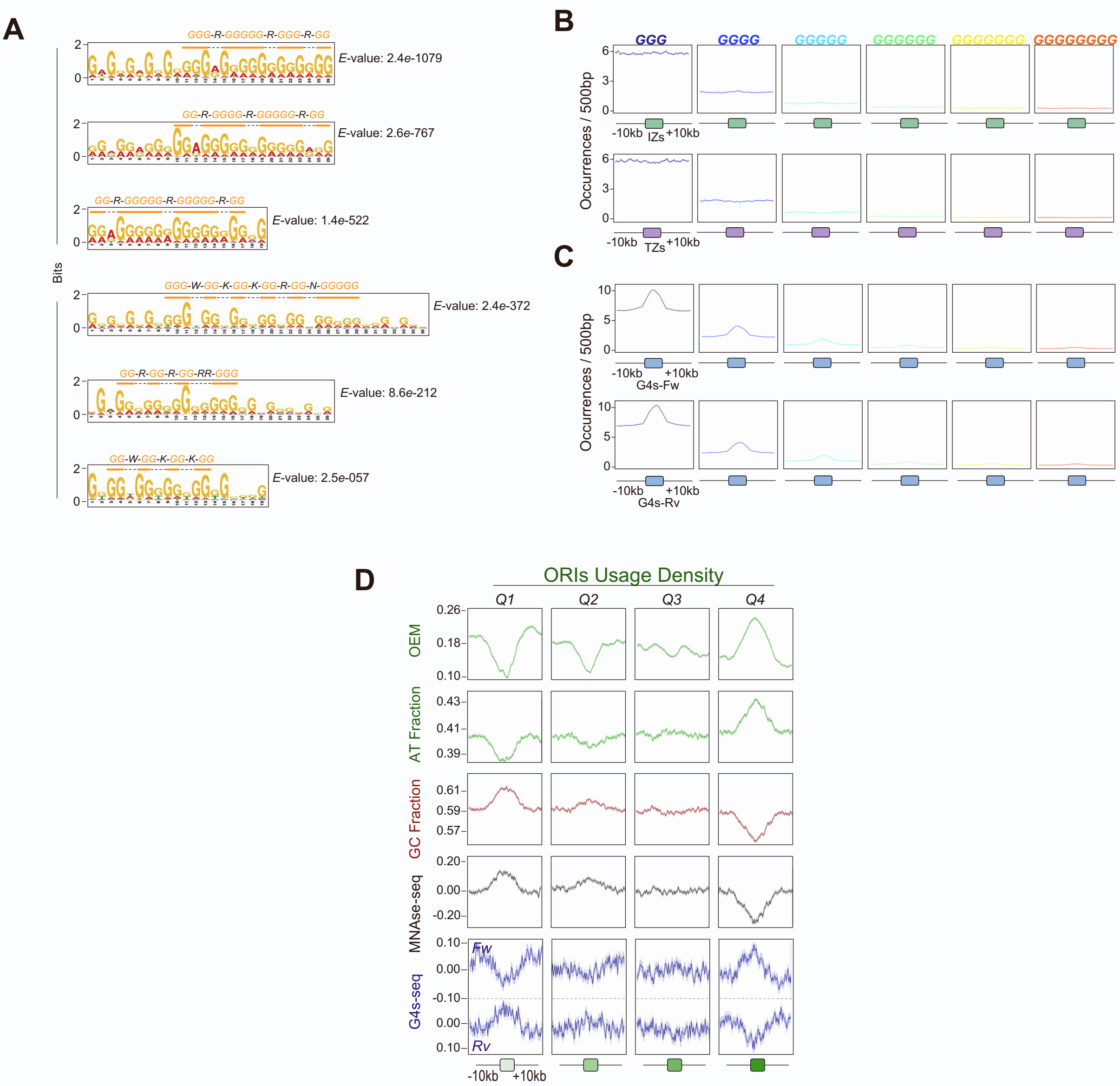

Figure S5. Sequence and chromatin content analysis in replication initiation zines, related to Figure 5. A) MEME sequence motif analysis of G4s overlapping IZs. G4s sequences from each motif is shown at the top with G tetrads in yellow and loops in black. All identified motifs fall into the $G_{2+}L_{1-12}$ G4s category, detectable mainly upon stabilisation (*Marsico et al, 2019*). **B)** Metaplots showing global profiles of poly(dG:dC) of the indicated lengths around all IZs and TZs from NT cells. **C)** Same as in B, but around all G4s found within IZs. **D)** Metaplots showing global OEM, AT and GC content, MNase-seq and G4-seq profiles around genome bins (5kb) grouped according to their ORI usage density from NT cells (Q1, lower usage; Q4, higher usage).
